# Ferulic Acid Attenuates Heat Stress-Induced Hepatic and Intestinal Oxidative Stress and Cholesterol Metabolism Dysregulation in Juvenile Blunt Snout Bream (*Megalobrama amblycephala*)

**DOI:** 10.3390/ijms27020925

**Published:** 2026-01-16

**Authors:** Yan Lin, Xiangjun Leng, Linjie Qian, Linghong Miao, Xiaoqin Li, Wenqiang Jiang, Siyue Lu, Zhengyan Gu

**Affiliations:** 1 Key Laboratory of Freshwater Fisheries and Germplasm Resources Utilization, Ministry of Agriculture and Rural Affairs, Freshwater Fisheries Research Center, Chinese Academy of Fishery Sciences, Wuxi 214081, China; liny@ffrc.cn (Y.L.); jiangwenqiang@ffrc.cn (W.J.); lusiyue@ffrc.cn (S.L.); guzhengyan@ffrc.cn (Z.G.); 2 National Demonstration Center for Experimental Fisheries Science Education, College of Fisheries and Life Science, Shanghai Ocean University, Shanghai 201306, China; xjleng@shou.edu.cn (X.L.); xqli@shou.edu.cn (X.L.); 3 Wuxi Fisheries College, Nanjing Agricultural University, Wuxi 214081, China; qianlinjiejie@gmail.com

**Keywords:** heat stress, ferulic acid, cholesterol metabolism, intestinal microbiota, metabolites

## Abstract

Ferulic acid (FA) is a green feed additive. To investigate the molecular mechanisms by which FA attenuates heat stress-induced hepatic and intestinal oxidative stress, as well as cholesterol metabolism disorders in *Megalobrama amblycephala* (9.75 ± 0.04 g), individuals were fed diets supplemented with 0, 100, or 200 mg/kg FA for eight weeks, followed by exposure to heat stress at 34 °C for 48 h. The results indicated that FA supplementation reduced malondialdehyde levels and downregulation genes involved in inflammatory responses (e.g., interleukin-6), apoptosis (e.g., caspase 8), and endoplasmic reticulum stress (e.g., immunoglobulin binding protein) (*p* < 0.05), which collectively alleviated heat stress-induced hepatic and intestinal oxidative stress. FA supplementation increased the expression of ATP-binding cassette transporter A1, apolipoprotein A1, and liver X receptor α (*p* < 0.05), and restored liver and plasma TC levels to pre-stress levels (*p* < 0.05). Additionally, FA ameliorated the heat stress-induced dysbiosis of the intestinal microbiota and modulated the composition and abundance of metabolites in intestinal contents and plasma, some of which are associated with cholesterol metabolism. In conclusion, dietary FA can alleviate heat stress-induced hepatic and intestinal oxidative stress, maintain the stability of the intestinal microbiota and regulate metabolic profiles, and improve the cholesterol metabolism disorders caused by heat stress.

## 1. Introduction

Aquatic products contribute approximately 15% of the animal-derived protein and 6% of the total protein consumed globally and are widely appreciated for their unique flavor and high nutritional value [1]. The expansion of the aquaculture sector has been significant in recent years; however, challenges such as environmental stress, animal welfare concerns, and metabolic dysfunctions have increasingly constrained its sustainable development [2]. Fish are highly sensitive to changes in ambient temperature due to their continuous dependence on the aquatic environment. In summer, water temperatures frequently exceed 32 °C, surpassing the optimal thermal range for most warm-water freshwater fish, impairing growth, development, reproduction, and metabolic homeostasis, and ultimately resulting in physiological damage or mortality [3,4,5].

Water temperature has emerged as a critical environmental parameter due to the increasing effects of global warming, thereby making heat stress a central focus in aquatic animal research [5,6]. The intestine is a critical mediator of pathophysiological responses to heat stress, and the intestinal microbiota functions as an essential biological barrier that maintains intestinal homeostasis [7,8]. Studies on aquatic animals such as largemouth bass (*Micropterus salmoides*) (acute heat stress) and blunt snout bream (*Megalobrama amblycephala*) (long-term heat stress) have shown that heat stress causes damage to intestinal tissues, altering the microbial community composition and the composition of intestinal metabolites [9,10]. Metabolites derived from the intestinal microbiota are transported via the bloodstream to the liver, where they modulate hepatic metabolism, a process crucial for managing various diseases [11,12]. Metabolomic and intestinal microbiota analysis in largemouth bass and rabbits revealed that lipid metabolism pathways are affected by heat stress [13,14]. The impact of heat stress on cholesterol metabolism has been established in livestock and poultry species [15]. In summary, heat stress disrupts microbiota composition and metabolism, contributing to cholesterol metabolic dysfunction and systemic tissue damage.

Ferulic acid (FA), a widely distributed hydroxycinnamic acid derivative in plants, displays multiple physiological activity-modulating functions, including free radical scavenging, the enhancement of antioxidant capacity, antimicrobial and anti-inflammatory effects, lipid-lowering potential, and the ability to improve muscle flavor characteristics [16,17,18,19]. Research has shown that ferulic acid mitigates growth inhibition and muscle characteristics alterations caused by heat stress in pigs [20]. In aquaculture, ferulic acid decreases the abundance of intestinal microbiota associated with energy and lipid metabolism in zebrafish (*Danio rerio*) [21]. Ferulic acid also alleviates the decreased antioxidant and immune performance induced by heat stress in tilapia (*Oreochromis mossambicus*) [22]. Ferulic acid demonstrates lipid-regulating properties by significantly lowering triglyceride (TG) and total cholesterol (TC) levels in both blood and liver [23]. Furthermore, ferulic acid mitigates the decline in digestive enzyme activity and stabilizes intestinal microbiota in Nile tilapia exposed to oxidized fish oil [24]. These findings indicate that ferulic acid may contribute to the regulation of intestinal microbiota and lipid metabolism homeostasis in fish in response to heat stress.

*M*. *amblycephala* is a significant cyprinid fish known for its flavorful meat, rapid growth, and high economic value. Contemporary studies on heat stress in *M. amblycephala* have mainly focused on blood physiology and biochemistry, liver oxidative stress, and antioxidant and immune responses. Heat stress has been shown to induce hepatic lipid and glycogen accumulation, hepatocyte structural alterations, and mitochondrial dysfunction [25,26]. While heat stress is known to affect aquatic organisms, its specific effects on the intestinal microbiota, metabolic alterations, and lipid homeostasis disruption in *M. amblycephala* have not been systematically investigated. Previous studies have reported that feed additives such as emodin, vitamin C, fructo-oligosaccharides, and folic acid can alleviate heat stress in *M. amblycephala*, although the selection was mainly based on antioxidant, immune, and endocrine functions [27,28]. Our research aims to investigate the effects of ferulic acid on heat stress-induced hepatic and intestinal oxidative stress and cholesterol metabolism disruption, and to elucidate the involvement and regulation of intestinal microbiota and metabolites in *M. amblycephala*.

## 2. Results

### 2.1. Effects of Dietary Ferulic Acid on Heat Stress-Induced Hepatic and Intestinal Oxidative Stress in M. amblycephala

Compared with the Control group, the activities of plasma alanine aminotransferase (ALT) and aspartate aminotransferase (AST) were significantly elevated in the S-Control group (*p* < 0.05), and there was no significant difference between the S-FA100 group and Control group (*p* > 0.05). In terms of antioxidant parameters, the S-Control group significantly increased hepatic malondialdehyde (MDA) content and decreased the glutathione reductase (GR) and glutathione S-transferase (GST) activities of *M. amblycephala* compared to the Control group (*p* < 0.05). However, MDA levels in the S-FA100 and S-FA200 groups, as well as the GST activity in the S-FA100 group, showed no significant difference compared to the Control group (*p* > 0.05). Heat stress had no significantly effect on intestinal MDA content or the activities of GR and GST (*p* > 0.05). However, intestinal MDA content was significantly lower in the S-FA100 group than that in S-Control group, and GR activity was significantly higher in the S-FA100 group than that in other groups (*p* < 0.05) (Table 1).

**Figure 1:**
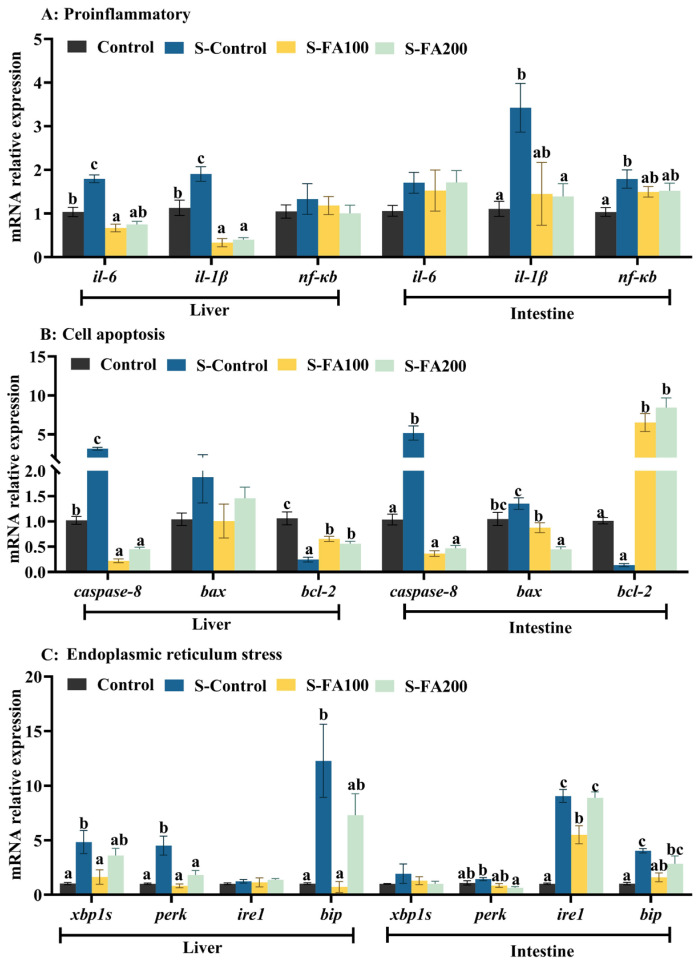
Effects of ferulic acid on hepatic and intestinal genes expression related to inflammation, apoptosis and endoplasmic reticulum stress in *M. amblycephala* under heat stress. (**A**) Expression levels of genes related to liver and intestinal inflammation. (**B**) Expression levels of genes related to liver and intestinal cell apoptosis. (**C**) Expression levels of genes related to liver and intestinal endoplasmic reticulum stress. Different lowercase letters on the different groups indicate significant differences between groups (Tukey’s test, *p* < 0.05). Data are shown as mean ± SEM (n = 3). *il-6*, interleukin-6; *il-1β*, interleukin-1β; *nf-κb*, nuclear transcription factor κB; *bax*, bcl-2 associated X gene; *bcl-2*, B-cell lymphoma-2; *xbp1s*, X-box-binding protein 1S; *perk*, protein kinase R-like ER kinase; *ire1*, inositol requiring enzyme 1; *bip*, immunoglobulin binding protein.

Compared with the Control group, the S-Control group showed significant upregulation of interleukin-6 (*il-6*) and interleukin-1β (*il-1β*) expression in the liver (*p* < 0.05). In contrast, their expression levels in the S-FA100 group were significantly lower than those in both the S-Control and Control groups (*p* < 0.05). In the intestine, the expression levels of *il-1β* and nuclear factor κB (*nf-κb*) were higher in the S-Control group than that in the Control group (*p* < 0.05). Furthermore, *il-1β* and *nf-κb* expression levels shown no significant differences between the S-FA100 or S-FA200 groups and the Control group in the intestine (*p* > 0.05) (Figure 1A).

Compared with the Control group, the S-Control group significantly upregulated the *caspase-8* expression level in the liver and intestine, and significantly downregulated the B-cell lymphoma 2 (*bcl-2*) expression level in the liver (*p* < 0.05). Furthermore, compared to the S-Control group, the *caspase-8* expression level was significantly lower and the *bcl-2* expression level was significantly higher in the liver and intestine of the S-FA100 and S-FA200 groups (*p* < 0.05), whereas the Bcl-2-associated X protein (*bax*) was significantly downregulated in the intestine (Figure 1B).

The expression levels of endoplasmic reticulum (ER) stress-related genes X-box binding protein 1S (*xbp1s*), protein kinase R-like ER kinase (*perk*), and immunoglobulin binding protein (*bip*)) in liver and inositol-requiring enzyme 1 (*ire1*) and *bip* in intestine were significantly increased in the S-Control group compared to the Control group (*p* < 0.05). In contrast, the expression of ER stress-related genes in the S-FA100 and S-FA200 groups showed no significant differences compared to the Control group in the liver (*p* > 0.05). However, in the intestine, the S-FA100 group significantly downregulated the *ire1* and *bip* expression levels compared to S-Control group (*p* < 0.05) (Figure 1C).

### 2.2. Effects of Dietary Ferulic Acid on Heat Stress-Induced Cholesterol Metabolism Dysregulation in M. amblycephala

**Figure 2:**
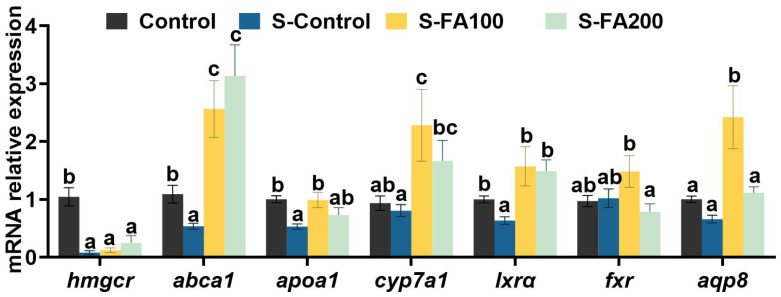
Effects of ferulic acid on heat stress-induced cholesterol metabolism genes expression of *M. amblycephala*. Different lowercase letters on the different groups indicate significant differences between groups (Tukey’s test, *p* < 0.05). Data are shown as mean ± SEM (n = 3). *hmgcr*, 3-hydroxy-3-methylglutaryl coenzyme A reductase; *abca1*, ATP-binding transporter A1; *apoa1*, apolipoprotein A1; *cyp7a1*, cholesterol 7α-hydroxylase; *lxrα*, liver X receptors α; *fxr*, farnesoid X receptor; *aqp8*, aquporin-8.

As shown in Table 2, compared to the Control group, plasma TC levels were significantly increased, while hepatic TC levels were significantly decreased in the S-Control group (*p* < 0.05). In ferulic acid supplementation groups, the plasma TC content of the S-FA100 and S-FA200 groups, as well as hepatic TC content of the S-FA200 group, was not significantly different from that of the Control group (*p* > 0.05). Compared with the Control group, the S-Control group showed significant downregulation of hepatic 3-hydroxy-3-methylglutaryl coenzyme A reductase (*hmgcr*), ATP-binding cassette transporter A1 (*abca1*), apolipoprotein A1 (*apoa1*), and liver X receptor α (*lxrα*) expressions (*p* < 0.05). Compared to the S-Control group, *abca1*, *lxrα* and cholesterol 7α-hydroxylase (*cyp7α1*) expression levels were significantly upregulated in the S-FA100 and S-FA200 groups, as was *apoa1* expression level in the S-FA100 group (*p* < 0.05). Moreover, the S-FA100 group showed significant upregulation of hepatic *apoa1*, farnesoid X receptor (*fxr*) and aquaporin-8 (*aqp8*) expression relative to the S-Control group (*p* < 0.05) (Figure 2).

### 2.3. Effects of Dietary Ferulic Acid on Heat Stress-Induced Intestinal Microbiota Dysbiosis in M. amblycephala

**Figure 3:**
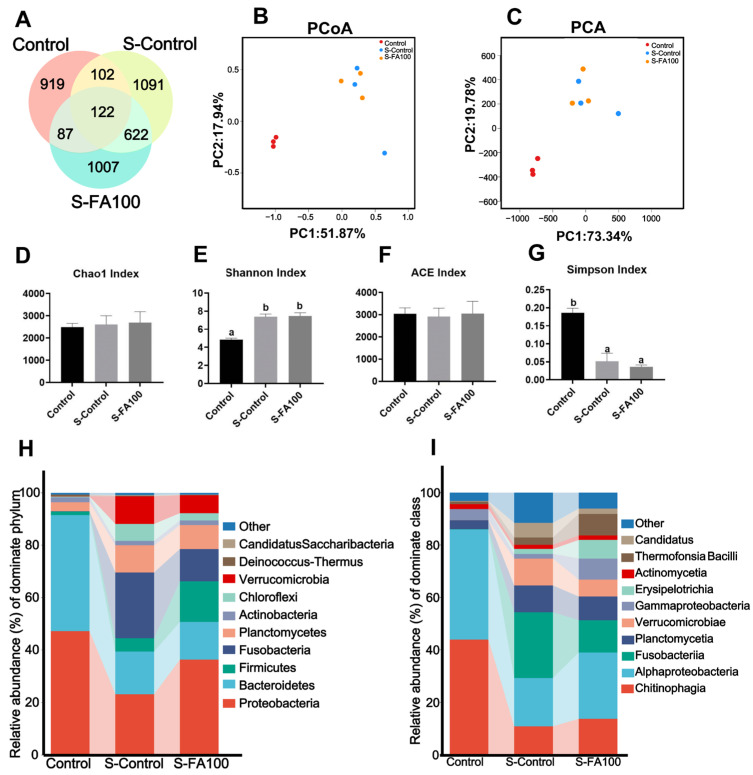
Analysis of intestinal microbiota of *M. amblycephala*. (**A**) OTU Venn analysis, distinct colors correspond to unique or shared the counts of OTUs. (**B**) OTU principal component analysis (PCoA analysis). (**C**) OTU principal coordinate analysis (PCA). (**D**–**G**) alpha diversity analysis. (**H**) Abundance of intestinal microbiota at the phylum level. (**I**) Abundance of intestinal microbiota at the class level. Different lowercase letters on the different groups indicate significant differences between groups (Tukey’s test, *p* < 0.05). Data are shown as mean ± SEM (n = 3).

A total of 121,840 valid sequences were obtained from nine pooled intestinal contents samples across the Control, S-Control, and S-FA100 groups, comprising 39,333 sequences from the Control group, 35,372 from the S-Control group, and 47,135 from the S-FA100 group. The average sequence length ranged from 1435 to 1447 bp (Table S2). Venn analysis identified 122 shared OTUs across the intestinal microbial communities in the Control, S-Control, and S-FA100 groups. Specifically, 102 OTUs were common between the Control and S-Control groups, 87 OTUs between the Control and S-FA100 groups, and 622 OTUs between the S-Control and S-FA100 groups. Unique OTUs numbered 919, 1091, and 1007 in the Control, S-Control, and S-FA100 groups, respectively (Figure 3A). PCA and PCoA revealed distinct separation between the intestinal microbiota of the Control and S-Control groups, whereas the microbiota composition of the S-FA100 group closely resembled that of the S-Control group following heat stress (Figure 3B,C). Alpha diversity metrics showed that the S-Control group displayed a significantly increased Shannon index and a significantly decreased Simpson index (*p* < 0.05) compared to the Control group. Chao1, Shannon, ACE, or Simpson indices showed no significant differences between the S-FA100 group and either the S-Control or Control groups (*p* > 0.05) (Figure 3D–G).

The compositional differences in intestinal microbiota at the phylum and class levels were further analyzed. Proteobacteria and Bacteroidetes constituted the predominant phylum in the Control group (Figure 3H). In the S-Control group, the relative abundances of Proteobacteria and Bacteroidetes were significantly reduced relative to the Control group (*p* < 0.05), while Verrucomicrobia showed a significant increase (*p* < 0.05) (Table 3). In the S-FA100 group, the abundances of Proteobacteria and Verrucomicrobia were restored to the Control group levels (*p* > 0.05) (Table 3). At the class level, Chitinophagia and Alphaproteobacteria were the two most abundant classes in the Control group (Figure 3I). The S-Control group showed a significant decrease in Chitinophagia and Alphaproteobacteria abundances, whereas Verrucomicrobiae abundance significantly increased compared to the Control group (*p* < 0.05). The abundances of Alphaproteobacteria and Verrucomicrobiae in the S-FA100 group returned to the Control group levels (*p* > 0.05) (Table 3).

### 2.4. Effects of Dietary Ferulic Acid on Heat Stress-Induced Changes in Intestinal Contents and Plasma Metabolome of M. amblycephala

**Figure 4:**
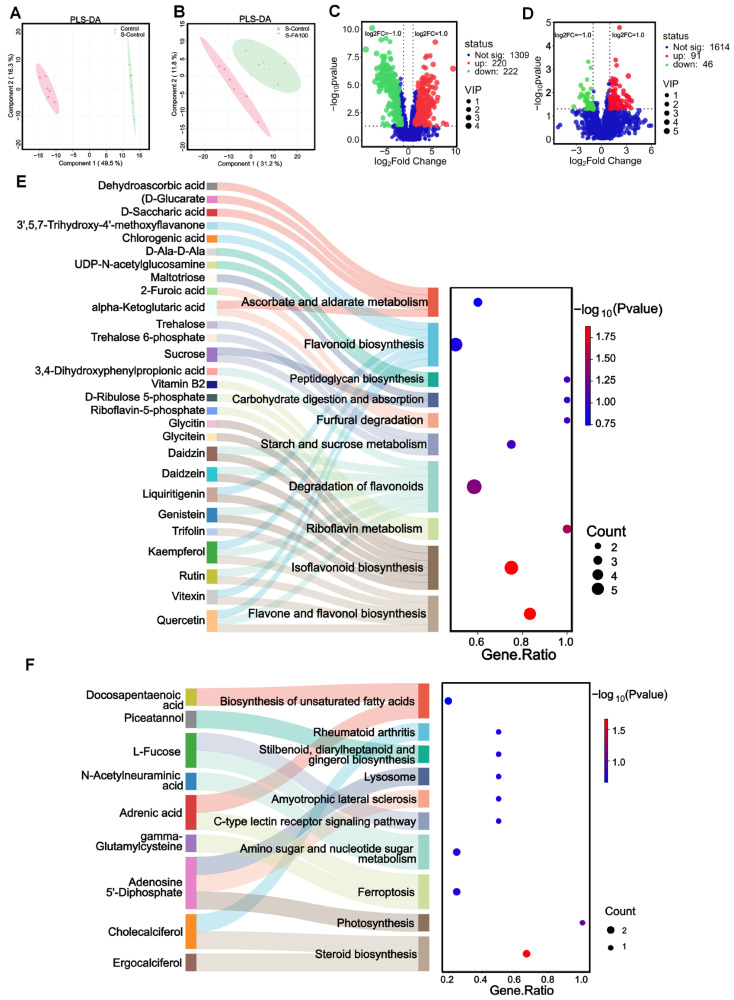
PLS-DA, volcano plots, and Sankey bubble plot of intestinal contents metabolome. (**A**,**C**,**E**) Metabolomic analysis of intestinal contents in S-Control vs. Control. (**B**,**D**,**F**) Metabolomic analysis of intestinal contents in S-FA100 vs. S-Control. (**A**,**B**) Partial least squares discriminant analysis (PLS-DA). (**C**,**D**) Volcano plots and red dots indicate differentially upregulated metabolites, green dots indicate differentially downregulated metabolites, and blue dots indicate no difference metabolites; differential metabolite screening threshold, VIP ≥ 1 & FDR-adjusted *p*-value ≤ 0.05 & |log2FC| ≥ 1; VIP, variable importance in the projection (VIP) values for the PLS-DA model; *p*-value, independent sample *t*-test for metabolite abundance; FC, Fold change mean abundance ratios of the groups. (**E**,**F**) Sankey bubble plot of KEGG metabolic pathway enrichment pathway of differential metabolites in intestinal contents; the horizontal axis represents the enrichment factor, the ratio of the number of differential metabolites enriched in a pathway to the background metabolites obtained by sequencing; the vertical axis represents the KEGG metabolic pathways enriched by differential metabolites (top 10 enrichment analysis statistical significance levels): the larger the circle, the larger the number of differential metabolites enriched to this metabolic pathways, and the larger the −log10 (*p*-value) value from red to green. Differential metabolites enriched in metabolic pathways are listed on the left, which are connected with the enriched metabolic pathways by colored bands. n = 3.

Metabolic profiles of intestinal contents from the Control, S-Control, and S-FA100 groups were analyzed by LC-MS to investigate metabolite changes induced by heat stress and ferulic acid treatment. PLS-DA analysis (Figure 4A,B) showed clear separation between the Control and S-Control groups, as well as between the S-Control and S-FA100 groups. A total of 442 differential metabolites were identified between the Control and S-Control groups, with 220 significantly upregulated and 222 significantly downregulated (Figure 4C, Table S4). Between the S-Control and S-FA100 groups, 137 differential metabolites were identified, including 91 upregulated and 46 downregulated ones (Figure 4D, Table S5).

**Figure 5:**
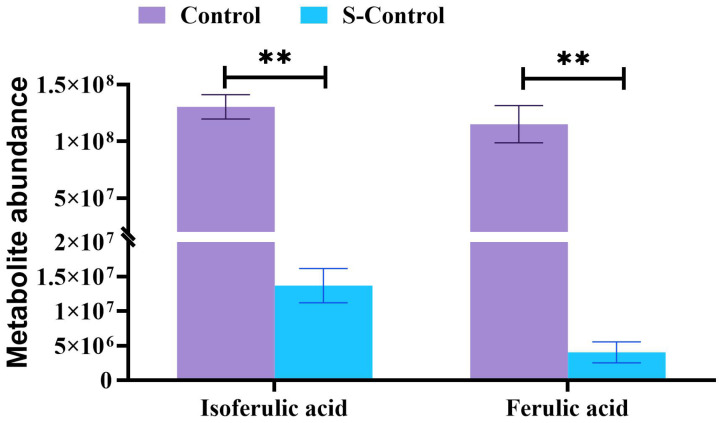
Differences in ferulic and isoferulic acid abundance in intestinal contents between S-Control and Control. “**” the differences were highly significant by independent sample *t*-test (*p* < 0.01). Data are shown as mean ± SEM (n = 3).

A Sankey plot illustrating the top 10 tertiary metabolic pathways based on KEGG enrichment significance was generated (Figure 4E,F). When comparing the S-Control group with the Control group, 28 differential metabolites were significantly enriched in flavone and flavonol biosynthesis, isoflavonoid biosynthesis, riboflavin metabolism, and other 7 pathways (Figure 4E). Downregulated metabolites such as quercetin, vitexin, rutin, kaempferol, and trifoliin were enriched in flavonoid and flavonol biosynthesis. Isoflavone biosynthesis pathway involved downregulated metabolites genistein, liquiritigenin, daidzein, daidzin, glycitein, and glycitin. Downregulated riboflavin-5-phosphate and vitamin B2, and upregulated D-ribulose-5-phosphate were enriched in the riboflavin metabolism pathway (Figure 4E, Table S4). Heat stress resulted in a substantial reduction in the levels of ferulic acid and isoferulic acid (*p* < 0.01, Figure 5).

When comparing the S-FA100 group with the S-Control group, 9 differential metabolites were enriched in steroid biosynthesis, photosynthesis, ferroptosis, and 7 other pathways (Figure 4F). The downregulated metabolites ergocalciferol and cholecalciferol were enriched in the steroid biosynthesis pathway; the photosynthesis pathway involved the upregulated metabolite adenosine 5′-diphosphate. Furthermore, the upregulated metabolite gamma-glutamylcysteine and the downregulated metabolite adrenic acid were enriched in the steroid biosynthesis pathway (Figure 4F, Table S5).

**Figure 6:**
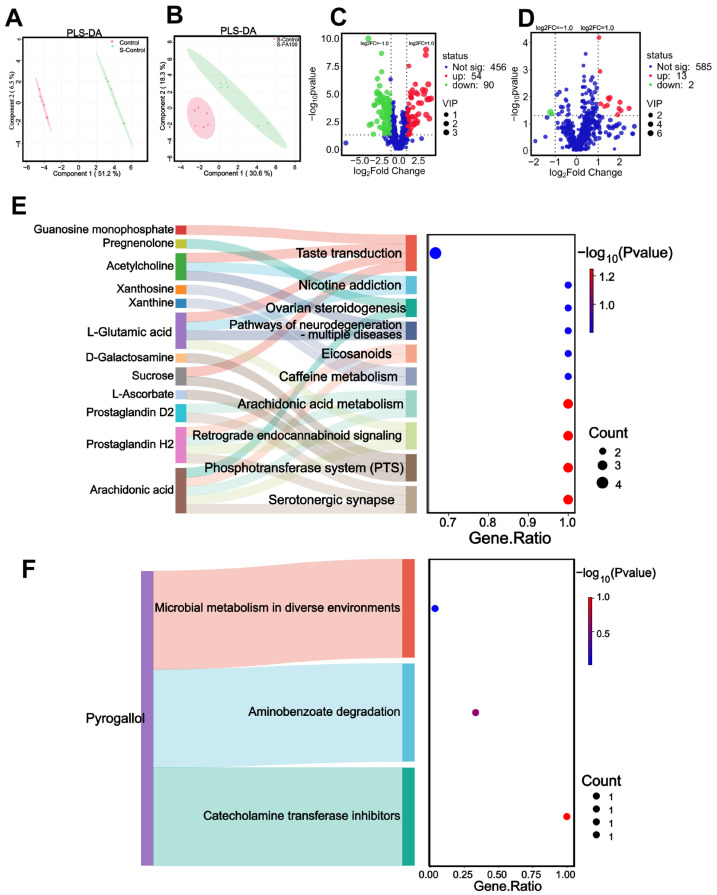
PLS-DA, volcano plots, and Sankey bubble plot of plasma metabolome. (**A**,**C**,**E**) Metabolomic analysis of plasma in S-Control vs. Control. (**B**,**D**,**F**) Metabolomic analysis of plasma in S-FA100 vs. S-Control. (**A**,**B**) Partial least squares discriminant analysis (PLS-DA). (**C**,**D**) Volcano plots: red dots indicate differentially upregulated metabolites, green dots indicate differentially downregulated metabolites, and blue dots indicate no difference metabolites; differential metabolite screening threshold, VIP ≥ 1 & FDR-adjusted *p*-value ≤ 0.05 & |log2FC| ≥ 1; VIP, variable importance in the projection (VIP) values for the PLS-DA model; *p*-value, independent sample *t*-test for metabolite abundance; FC, mean abundance ratios of the groups. (**E**,**F**) Sankey bubble plot of KEGG metabolic pathway enrichment pathway of differential metabolites in intestinal contents; the horizontal axis represents the enrichment factor, the ratio of the number of differential metabolites enriched in a pathway to the background metabolites obtained by sequencing; the vertical axis represents the KEGG third-level metabolic pathways enriched by differential metabolites (top 10 enrichment analysis statistical significance levels): the larger the circle, the larger the number of differential metabolites enriched to this metabolic pathways, and the larger the −log10 (*p*-value) value from red to green. Differential metabolites enriched in metabolic pathways are listed on the left, which are connected with the enriched metabolic pathways by colored bands. n = 3.

Plasma metabolic profiles of the Control, S-Control, and S-FA100 groups were analyzed by LC-MS to examine changes induced by heat stress and ferulic acid treatment. PLS-DA analysis revealed clear separation between the Control and S-Control groups, as well as between the S-Control and S-FA100 groups (Figure 6A,B).

A total of 144 differential metabolites were identified between the S-Control and Control groups, including 54 upregulated and 90 downregulated metabolites (Figure 6C, Table S6). A Sankey plot illustrating the top 10 tertiary metabolic pathways based on KEGG enrichment significance was generated (Figure 6E). Overall, 12 differential metabolites were mainly enriched in the serotonergic synapse, phosphotransferase system (PTS), retrograde endocannabinoid and other 7 related pathways. Notably, the downregulated arachidonic acid and prostaglandin H2 were enriched in pathways including serotonergic synapse, retrograde endocannabinoid, arachidonic acid metabolism, and eicosanoids pathways.

Between the S-FA100 and S-Control groups, 15 differential metabolites were identified, comprising 13 upregulated and 2 downregulated metabolites (Figure 6D, Table S7). A Sankey plot illustrating the top 10 tertiary metabolic pathways based on KEGG enrichment significance was generated (Figure 6F). Downregulated pyrogallol was enriched in the pathways of catecholamine transferase inhibitors, aminobenzoate degradation, and microbial metabolism in diverse environments (Figure 6F, Table S7).

**Figure 7:**
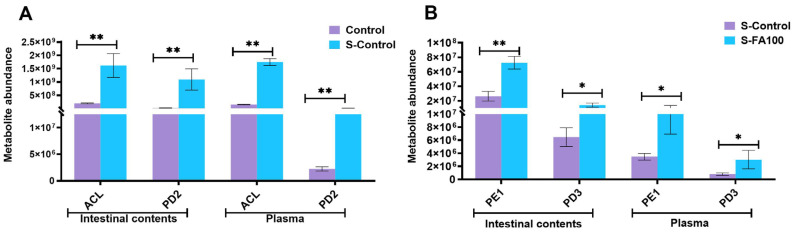
Metabolites are shared between intestinal contents and plasma. (**A**) S-Control vs. Control. (**B**) S-FA100 vs. S-Control. ACL, acetylcholine; PD2, prostaglandin D2; PD3, prostaglandin D3; PE1, 2,3-dinor prostaglandin E1. “*” indicates significant difference (*p* < 0.05, *t*-test), “**” indicates extremely significant difference (*p* < 0.01, *t*-test). Data are shown as mean ± SEM (Data are shown as mean ± SEM (n = 3).

Compared to the Control group, acetylcholine (ACL) and prostaglandin D2 (PD2) levels in both intestinal contents and plasma were significantly elevated in the S-Control group, demonstrating a consistent trend across the two sample types (*p* < 0.01, Figure 7A). Compared to the S-Control group, the S-FA100 group exhibited a marked increase in 2, 3-dinor prostaglandin E1 (PE1) and prostaglandin D3 (PD3) in both intestinal contents and plasma with changes that were consistent across the two biological matrices (*p* < 0.05, Figure 7B).

### 2.5. Correlation Analysis

**Figure 8:**
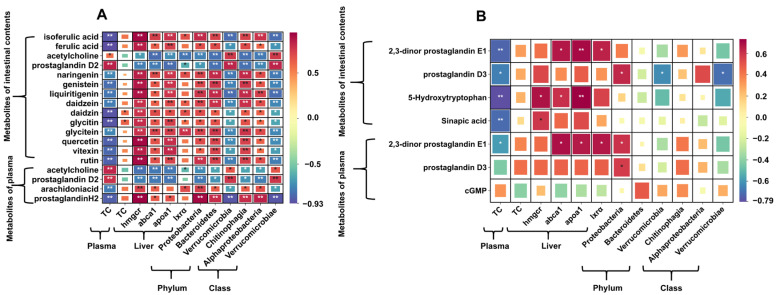
Correlation analysis of intestinal contents and plasma differential metabolites with the phylum and class of intestinal microbiota abundance and lipid metabolism indicators. (**A**) S-Control vs. Control. (**B**) S-FA100 vs. S-Control. The horizontal axis represents the plasma and liver related indicators, and intestinal contents and plasma differential metabolites are shown on the vertical axis. “*” indicates significant correlation (*p* < 0.05). “**” indicates extremely significant correlation (*p* < 0.01). Blue indicates negative correlation, red indicates positive correlation.

Spearman correlation analysis was conducted to evaluate the relationships between metabolites in intestinal contents and plasma, lipid metabolism indices, and differential microbial taxa at the phylum and class levels. Specifically, comparisons were made between the S-Control and the Control groups, as well as between the S-FA100 group and the S-Control group. As shown in Figure 8A, in the comparison between the S-Control and Control groups, metabolites upregulated in both plasma and intestinal contents, such as acetylcholine and prostaglandin D2, were positively correlated with plasma TC levels and negatively correlated with the expression of cholesterol metabolism genes (*hmgcr*, *abca1*, *apoa1*, and *lxrα*) (*p* < 0.05). Ferulic acid and isoferulic acid, which were significantly decreased by heat stress, showed significant negative correlations with plasma TC, Verrucomicrobia, and Verrucomicrobiae, while displaying positive correlations with cholesterol metabolism gene expression as well as Proteobacteria, Bacteroidetes, Chitinophagia, and Alphaproteobacteria (*p* < 0.05). Similarly, downregulated metabolites, including naringenin, genistein, and liquiritigenin in intestinal contents, as well as arachidonic acid and prostaglandin H2 in plasma, showed correlation patterns similar to those observed for ferulic acid and isoferulic acid (*p* < 0.05).

The correlation analysis between variables in the S-FA100 and S-Control groups (Figure 8B). The metabolites PE1 and PD3, both upregulated in plasma and intestinal contents, showed distinct associations: PE1 showed a significant positive correlation with cholesterol metabolism-related gene expression levels (*p* < 0.05), whereas PD3 was significantly positively associated with the Proteobacteria abundance (*p* < 0.05). Furthermore, 5-Hydroxytryptophan, an upregulated metabolite in intestinal contents, showed a significant positive correlation with cholesterol metabolism gene expression levels (*p* < 0.05). Intestinal PD3 was significantly negatively correlated with plasma TC levels and the abundance of Verrucomicrobia and Verrucomicrobiae (*p* < 0.05).

## 3. Discussion

Ferulic acid has been utilized as a feed additive in aquaculture. Our previous research demonstrated that supplementing feed with 100 and 200 mg/kg of ferulic acid improved the growth performance, enhanced the hepatic and intestinal antioxidant capacity, and alleviated ER stress and inflammatory responses in *M. amblycephala* [29]. The present study further investigated the protective function of ferulic acid against heat stress.

### 3.1. Dietary Ferulic Acid Attenuates Heat Stress-Induced Hepatic and Intestinal Oxidative Stress in M. amblycephala

Elevated ALT and AST in plasma indicate liver damage [30]. In our study, heat stress significantly elevated plasma ALT and AST levels, whereas ferulic acid supplementation effectively mitigated these changes. Specifically, 100 mg/kg ferulic acid restored ALT and AST activities to pre-stress levels, indicating protection against heat stress-induced liver injury [31]. These findings align with previous results demonstrating that dietary ferulic acid reduced plasma ALT in *M. amblycephala* [29]. Furthermore, increased MDA levels in liver and intestinal tissues confirmed oxidative stress induced by heat stress, which were attenuated by ferulic acid supplementation, with levels restored to pre-stress conditions [32]. Heat stress impairs hepatic antioxidant function primarily through the suppression of GR and GST activities, whereas ferulic acid counteracted these effects by enhancing GR and GST activities, thereby supporting hepatic antioxidant defense [29]. It also mitigates LPS-induced hepatic oxidative damage by upregulating these enzymes [33]. Several studies have further confirmed that ferulic acid increases antioxidant status in fish species [22]. Furthermore, a dose of 100 mg/kg ferulic acid was more effective than 200 mg/kg in improving heat stress tolerance in *M. amblycephala*.

Ferulic acid has been reported to exhibit anti-inflammatory effects through the suppression of proinflammatory cytokine expression [34]. In the context of heat stress, ferulic acid significantly reduced the expressions of *il-6*, *il-1β*, and *nfκb* in liver and intestine, indicating its capacity to attenuate heat stress-induced tissue inflammation [35,36,37]. Oral administration of ferulic acid has also been shown to alleviate LPS-induced liver inflammation in *M. amblycephala* by decreasing hepatic *il-1β* expression level [33], and reduce neuroinflammation in mice by downregulating *il-6* and *il-1β* expression levels [38]. The suppression of intestinal *nf-κb* may play a pivotal role in limiting *il-6* expression, preventing the dysregulation of inflammatory mediators and the development of inflammation-associated pathologies [39].

Heat stress has been shown to activate *caspase-8* and inhibit *bcl-2* expressions in both liver and intestine, thereby promoting apoptosis in these tissues in the present study. Supplementation with 100 and 200 mg/kg ferulic acid attenuated the dysregulation of apoptosis-related gene expression. Similar findings have been reported in mice [40], *M. amblycephala* [29], and hybrid grouper (*Epinephelus fuscoguttatus*♀ × *Epinephelus polyphekadion*♂), supporting the role of ferulic acid in regulating apoptosis through the modulation of apoptosis-associated gene expressions [41]. More evidence further suggests that the anti-apoptotic effects of ferulic acid are likely mediated via the suppression of the NF-κB signaling pathway [42].

ER stress, triggered by the accumulation of unfolded or misfolded proteins, has been implicated in tissue injury [43,44]. Ferulic acid has been reported to alleviate ER stress, thereby exerting cardioprotective and neuroprotection effects against ER stress-induced neuronal death [45,46]. In the present study, heat stress markedly activated ER stress-related genes, including *xbp1s*, *perk*, and *bip* in the liver, as well as *perk*, *ire1*, and *bip* in the intestine, reflecting the disruption of ER homeostasis. Dietary ferulic acid supplementation effectively counteracted these alterations, mitigating heat stress-induced ER stress in both liver and intestine. Collectively, these findings suggest that ferulic acid exerts protective effects under heat stress by attenuating inflammation, apoptosis, and ER stress in the liver and intestine.

### 3.2. Dietary Ferulic Acid Attenuates Heat Stress-Induced Cholesterol Metabolism Dysregulation in M. amblycephala

Previous studies have shown that supplementing the feed with ferulic acid can alleviate oxidative stress, inflammatory response, cell apoptosis and endoplasmic reticulum stress in the liver and intestines of *M. amblycephala* caused by heat stress. However, heat stress significantly influences lipid metabolism in fish and other animals [47,48]. Therefore, it is important to investigate the effect of ferulic acid on lipid metabolism under heat stress conditions. Cholesterol, a vital lipid constituent, is essential for maintaining cell membrane structure and function, regulating membrane fluidity, and counteracting heat-induced membrane destabilization, thereby preserving cellular integrity [49,50]. In this study, heat stress led to an abnormal elevation of plasma TC levels accompanied by a marked decrease in hepatic TC. The downregulation of *hmgcr*, a key gene in cholesterol biosynthesis [51], likely contributes to the reduction in hepatic cholesterol. This imbalance was further supported by decrease hepatic expressions of *apoa1* and *abca1*, suggesting impaired cholesterol transport to the liver [52,53]. APOA1, together with ABCA1, mediates the efflux of free cholesterol and phospholipids, promoting the formation of high-density lipoprotein, which subsequently delivers cholesteryl esters to the liver for metabolism [54,55]. A similar pattern was reported in pigs, where acute heat stress suppressed hepatic cholesterol synthesis but elevated serum cholesterol levels. Furthermore, *lxrα*, a transcription factor that upregulates *apoa1* expression, was significantly downregulated in the liver under heat stress, thereby further disrupting cholesterol transport and metabolism [56,57]. Upon activation, LXRα forms a heterodimer with retinoid X receptor (RXR) and binds to the ABCA1 promoter, initiating its transcription. ABCA1 facilitates APOA1 lipidation, thereby enhancing reverse cholesterol transport [58,59]. In present study, heat stress inhibited *lxrα*, *abca1* and *apoa1* expressions, thereby impairing reverse cholesterol transport, and leading to plasma cholesterol accumulation. Ferulic acid alleviated the adverse effects of heat stress. Ferulic acid supplementation restored the heat stress-induced dysregulation of *apoa1*, *abca1*, and *lxrα* expression, which in turn normalized both plasma and hepatic TC levels. These findings suggest that dietary ferulic acid can effectively mitigate heat stress-induced dysregulation of cholesterol metabolism by modulating key genes involved in cholesterol biosynthesis and transport.

### 3.3. Dietary Ferulic Acid Attenuates Heat Stress-Induced Intestinal Microbiota Dysbiosis in M. amblycephala

Intestinal microbial communities critically influence host metabolism, systemic health, and behavioral outcomes through complex host–microbe interactions, primarily via microbially derived metabolites, hormones, and neurotransmitters [60]. A close functional connection exists between the intestine and liver, in which microbial metabolites are transported via the bloodstream to the liver, influencing hepatic metabolism [11,12]. Heat stress alters the composition and diversity of intestinal microbiota in fish [61]. In rainbow trout, heat stress induces dysbiosis and shifts in intestinal metabolites, particularly disrupting lipid and amino acid metabolism [14]. In tilapia, ferulic acid ameliorated microbial imbalances caused by oxidized fish oil and heat stress [22,23,24]. In the present study, heat stress significantly reduced the relative abundances of Proteobacteria and Alphaproteobacteria, whereas it markedly increased the abundances of Verrucomicrobia and Verrucomicrobiae. However, supplementation with ferulic acid restored the levels of Proteobacteria, Verrucomicrobia, Alphaproteobacteria, and Verrucomicrobiae to those observed in the Control group. These findings indicate that ferulic acid can partially ameliorate intestinal microbiota dysbiosis induced by heat stress. Studies on medaka (*Oryzias melastigma*) demonstrated that exposure to naphthenic acids caused intestinal damage, inflammation and oxidative stress, characterized by decreased Proteobacteria and increased Verrucomicrobia abundance in the high-concentration exposure group [62]. Consistent with our findings, intestinal Proteobacteria and Verrucomicrobia exhibited a similar trend of change after stress exposure. Studies in pigs revealed that fermented feed enhances intestinal immune performance, and decreased the abundance of Verrucomicrobia [63]. These findings point to a potential connection between the imbalance of Proteobacteria and Verrucomicrobia and the health of the organism. The homeostasis of intestinal microbiota in fish plays a crucial role in maintaining the health of the organism and regulating nutrient metabolism [64]. Therefore, the findings indicate that dietary ferulic acid mainly maintains the homeostasis of Proteobacteria and Verrucomicrobia to cope with the intestinal microbiota disorder in *M. amblycephala* caused by heat stress.

### 3.4. Dietary Ferulic Acid Attenuates Heat Stress-Induced Changes in Intestinal Contents and Plasma Metabolome of M. amblycephala

Heat stress disrupts the intestinal microbiota of *M. amblycephala*, which may lead to subsequent perturbations in intestinal metabolites. This potential cascade warrants further investigation. Recent studies have systematically characterized the effects of heat stress on hepatic metabolite profiles using metabolomic approaches [48,65]. In Sebastes schlegelii, plasma metabolome analysis found that acute heat stress significantly altered metabolites associated with energy, amino acid, and fatty acid metabolism [66]. The present study demonstrated that both heat stress and dietary supplementation with ferulic acid influenced the composition of metabolites in intestinal contents and plasma. Heat stress decreased levels of ferulic acid and isoferulic acid in the intestinal contents, suggesting that exogenous supplementation may enhance thermotolerance in *M. amblycephala*. Metabolomic profiling of intestinal contents under heat stress revealed significant enrichment of four metabolic pathways: isoflavonoid biosynthesis, flavone and flavonol biosynthesis, riboflavin metabolism, and flavonoid biosynthesis. Key metabolites involved in these pathways, including naringenin, genistein, glycyrrhizin, daidzein, quercetin, vitexin, and rutin, were significantly downregulated. These flavonoids are known to modulate lipid metabolism, suppress inflammatory responses, and contribute to systemic physiological homeostasis [67,68]. In plasma, heat stress-induced differential metabolites, such as arachidonic acid and prostaglandin H2, were primarily associated with serotonergic synapse, retrograde endocannabinoid signaling, arachidonic acid metabolism, platelet activation, and oxytocin signaling pathways. Furthermore, increased concentrations of acetylcholine and prostaglandin D2 were observed in both intestinal contents and plasma under heat stress. Acetylcholine is implicated in tumorigenic processes [69], while prostaglandin D2 is a known proinflammatory mediator [70], indicating their potential roles in heat stress-induced hepatic and intestinal oxidative stress. Correlation analysis further suggested that these metabolites may contribute to elevated plasma TC levels and disturbances of cholesterol metabolism.

In comparison to the S-Control group, levels of PE1 and PD3 in both intestinal contents and plasma were significantly elevated in the S-FA100 group, showing consistent patterns between intestinal contents and plasma. The PE1 in intestinal contents and plasma, along with 5-hydroxytryptophan in intestinal contents, was negatively correlated with plasma TC and positively correlated with the expression levels of genes regulating cholesterol metabolism. Although research on PE1 has primarily focused on its therapeutic role in promoting angiogenesis [71], the present findings indicate a potential link to lipid regulation. PD3 in intestinal content was negatively correlated with plasma TC and the abundance of Verrucomicrobia and Verrucomicrobiae, while positively correlated with Proteobacteria. Given its previously reported antithrombotic properties [72], PD3 may also participate in modulating lipid metabolism and microbial composition. Previous studies have shown that the improvement of intestinal microflora in *M*. *amblycephala* can promote cholesterol metabolism and reduce lipid deposition [73].

## 4. Materials and Methods

### 4.1. Feed and Experimental Design

Soybean meal, rapeseed meal, cottonseed meal, and cottonseed protein concentrate were used as the primary protein sources. Soybean oil and wheat flour served as lipid source and carbohydrate source, separately. Three diets were prepared with ferulic acid (Sigma Aldrich, Shanghai; purity = 99%) supplementation at concentrations of 0, 100, and 200 mg/kg. All diets were isonitrogenous and isoenergetic. The ingredients were processed into uniform pellets with a 1.5 mm diameter using an F-26 (II) granulator, followed by air-drying and storage at −20 °C. Feed formulation is shown in Table S1.

### 4.2. Fish, Feeding Trial and Heat Stress Treatment

*M. amblycephala* (Huahai No.1, initial weight = 9.75 ± 0.04 g) juveniles were acclimated for 15 days at the Nanquan farming base of FFRC, CAFS. A total of 180 healthy and size-uniform juveniles were randomly assigned to nine glass tanks (0.6 m × 0.8 m × 0.8 m) in a recirculating aquaculture system, with three replicates (20 fish/tank) per treatment. Fish were fed diets supplemented with 0, 100, or 200 mg/kg ferulic acid (Control, FA100, FA200) three times daily (07:30–08:00, 11:30–12:00, and 17:00–17:30) for 8 weeks. During the feeding period, the fish were fed excessively, and any uneaten feed was removed 30 min after feeding. Tanks were maintained under natural photoperiod and continuous aeration, with weekly water exchange (25%) and monitoring (DO ≥ 6.0 mg/L, 27 ± 0.5 °C, total ammonia < 0.05 mg/L, nitrite < 0.01 mg/L, pH 6.8–7.5). Temperature and water quality were controlled as described in our group’s previous publication [17].

After the 8-week feeding trial, three fish per replicate were sampled from the Control group, and 16 fish per tank from all groups were subjected to heat stress testing. Based on the preliminary experimental results of our research group and the studies on *M. amblycephala* response to heat stress [74,75], water temperature was raised from 27 °C to 34 °C at a rate of 2 °C/day. Fish samples were collected after maintaining the temperature for 48 h. Feeding continued during heating and stabilization. Following heat stress exposure, the groups were designated as S-Control, S-FA100, and S-FA200. Water quality, except temperature, was maintained within the same ranges as above. No mortality occurred during the trial.

### 4.3. Sample Collection

The Control (pre-stress), S-Control, S-FA100, and S-FA200 groups were subjected to a 12 h fasting period prior to sample collection. Fish from each tank were immediately subjected to deep anesthesia using MS-222 (200 mg/L). Subsequently, three fish were randomly selected from each tank, and plasma collection was performed as described in a previous study [76]. Plasma samples were divided into two portions and stored at −80 °C for subsequent biochemical analysis and metabolome profiling. The intestinal contents of per fish were divided into two portions and stored at −80 °C for the analysis of microbiota and metabolome analyses. Liver and intestine tissues were rapidly dissected from three fish per tank for the evaluation of antioxidant parameters and gene expression analysis.

### 4.4. Analyses of Indicators

#### 4.4.1. Plasma Biochemical Indices

The activities of ALT (ultraviolet-lactate dehydrogenase method) and AST (ltraviolet-malate dehydrogenase method), as well as the TC (COD-PAP method) level in plasma, were detested using commercial kits (Mindray Biomedical Co., Ltd., Shenzhen, China) and an automated biochemical analyzer (Mindray BS-400, Shenzhen, China). The detailed measurement method was strictly carried out in accordance with the instructions.

#### 4.4.2. Antioxidant Indexes and Liver TC Content

Liver and intestinal tissue samples (0.1 g per serving) were homogenized in 0.9 mL of normal saline to prepare a 10% (*w*/*v*) homogenate. The homogenate was centrifuged at 6000 rpm for 10 min. Subsequently, the supernatant was collected and stored at −20 °C for the determination of relevant indicators. MDA (A003-1-2, TBA method) content, and activities of GR (A062-1-1, Ultraviolet spectrophotometry method) and GST (A0041-1, Colorimetry) in the liver and intestine, as well as TC (A111-1-1, COD-PAP method) content in the liver, were determined using commercial kits from Nanjing Jiancheng Bioengineering Institute (Nanjing, China). The detailed measurement method was strictly carried out in accordance with the instructions.

#### 4.4.3. RNA Extraction and Real-Time Fluorescence Quantitative PCR (qRT-PCR) Analysis

Total RNA was extracted from liver tissues, and qRT-PCR analysis was conducted as previously described protocol [75]. Primers targeting *il-6*, *il-1β*, *nf-κb*, *caspase-8*, *bax*, *bcl-2*, *xbp1s*, *perk*, *ire1*, *bip*, *hmgcr*, *abca1*, *apoa1*, *cyp7α1*, *lxrα*, *fxr*, and *aqp8* were designed using NCBI Primer-Blast and synthesized by Shanghai Sangon Biotechnology Co., Ltd. (Shanghai, China) (Table S2). Gene expression levels were normalized to beta-cytoskeletal actin (*β-actin*), and relative mRNA quantification was calculated using the 2^−ΔΔCt^ method.

#### 4.4.4. Analysis of Intestinal Microbiota

The intestinal contents from three fish per tank were pooled in a 1:1:1 ratio, and three mixed samples from each group were subjected to intestinal microbiota analysis. The intestinal microbiota composition of *M. amblycephala* juveniles was assessed using 16S rRNA metagenomic sequencing, following the methodology described by [77]. The circular consensus sequencing (CCS) of PacBio data was performed using SMRTLINK (v9). Operational taxonomic units (OTUs) were clustered at 98.65% sequence similarity, with chimeric sequences removed. Taxonomic classification of OTU representative sequences was performed using a clustering algorithm, characterizing community composition at the phylum and class levels. Alpha diversity was quantified using Chao1, ACE, Shannon, and Simpson indices. Beta diversity was assessed by calculating Bray–Curtis dissimilarity between pairwise samples. Principal coordinates analysis (PCoA) and principal component analysis (PCA) were conducted to visualize β-diversity within microbial communities. The detailed steps of the testing methods are described in the supplementary document, “Supplementary Explanation”.

#### 4.4.5. Metabolome Analysis of Intestinal Contents and Plasma

Liquid chromatography–mass spectrometry (LC-MS) was used to identify and quantify metabolites in intestinal contents and plasma samples. The metabolome analysis of intestinal contents and plasma was conducted using samples from two fish per tank, with the detection method described in detail in the supplementary file “Supplementary Explanation”. Data analysis was performed using partial least squares-discriminant analysis (PLS-DA). Differentially expressed metabolites were identified based on the criteria: VIP ≥ 1, FDR-adjusted *p*-value ≤ 0.05, and |log2FC| ≥ 1. Here, VIP refers to the Variable Importance in Projection values derived from the PLS-DA model; the *p*-value is derived from a *t*-test comparing metabolite abundances between groups; and FC represents the fold change in mean metabolite abundance. Furthermore, KEGG pathway analysis was conducted to identify metabolic pathways associated with the differential metabolites, followed by enrichment analysis to localize the pathways involved.

### 4.5. Statistics and Analysis

Kolmogorov–Smirnov test and Levene’s test in SPSS (v 20.0) are used to analyze the normality assessment and homogeneity of variance. Statistical comparisons were performed using one-way ANOVA (Tukey’s multiple comparison tests), with *p* < 0.05 indicating mean significant difference. Independent sample *t*-tests were conducted to analyze intestinal microbiota abundance and metabolite differences, with significance defined at *p* < 0.05. Data are shown as mean ± standard error of means (x ± SEM).

## 5. Conclusions

In conclusion, while heat stress decreased the ferulic acid and isoferulic acid levels in the intestinal contents, dietary ferulic acid supplementation mitigated heat stress-induced hepatic and intestinal oxidative stress and reduced plasma TC accumulation. This protective effect was associated with the upregulation of key genes involved in cholesterol metabolism, including *apoa1*, *abca1*, and *lxrα*. Moreover, ferulic acid alleviated heat stress-induced dysbiosis and restored intestinal microbial homeostasis and metabolite profiles in both intestinal contents and plasma. This study provides a reference for the healthy breeding of *M. amblycephala* and the basis on heat stress in aquatic animals.

## Figures and Tables

**Table 1:** Effects of ferulic acid on the hepatic and intestinal antioxidant properties and liver injury indicators in *M. amblycephala* under heat stress.

Types	Items	Control	S-Control	S-FA100	S-FA200
Plasma	ALT, U/L	2.6 ± 0.13 ^a^	193.5 ± 10.67 ^c^	11.6 ± 3.06 ^a^	65.0 ± 4.61 ^b^
	AST, U/L	58.42 ± 3.03 ^a^	293.0 ± 6.80 ^b^	105.12 ± 22.82 ^a^	274.5 ± 13.17 ^b^
Liver	MDA, mmol/mgprot	1.55 ± 0.08 ^a^	2.191 ± 0.14 ^b^	1.41 ± 0.16 ^a^	1.67 ± 0.15 ^ab^
	GR, U/mgprot	4.98 ± 0.16 ^c^	1.91 ± 0.25 ^a^	3.60 ± 0.33 ^b^	3.75 ± 0.30 ^b^
	GST, U/mgprot	20.72 ± 0.70 ^c^	8.55 ± 1.53 ^a^	20.43 ± 1.29 ^c^	15.71 ± 1.36 ^b^
Intestine	MDA, mmol/mgprot	5.34 ± 0.19 ^ab^	6.69 ± 0.32 ^b^	4.73 ± 0.52 ^a^	5.37 ± 0.42 ^ab^
	GR, U/mgprot	14.67 ± 0.89 ^a^	14.22 ± 0.75 ^a^	22.50 ± 2.28 ^b^	15.25 ± 1.55 ^a^
	GST, U/mgprot	61.60 ± 6.45	47.94 ± 4.95	53.81 ± 4.05	55.31 ± 4.53

Different lowercase letters on the different groups indicate significant differences between groups (Tukey’s test, *p* < 0.05). Data are shown as mean ± SEM (n = 3). ALT, alanine aminotransferase; AST, aspartate aminotransferase; MDA, malondialdehyde; GR, glutathione reductase; GST, glutathione S-transferase.

**Table 2:** Effects of ferulic acid on heat stress-induced total cholesterol changes in the liver and plasma of *M. amblycephala*.

Types	Items	Control	S-Control	S-FA100	S-FA200
Plasma	TC, mmol/L	6.41 ± 0.10 ^a^	7.13 ± 0.17 ^b^	6.41 ± 0.15 ^a^	6.04 ± 0.18 ^a^
Liver	TC, mmol/L	56.00 ± 3.00 ^b^	33.0 ± 5.20 ^a^	33.8 ± 3.30 ^a^	50.9 ± 7.10 ^ab^

Different lowercase letters on the different groups indicate significant differences between groups (Tukey’s test, *p* < 0.05). Data are shown as mean ± SEM (n = 3). TC, total cholesterol.

**Table 3:** Significant differences phylum and class among the top 10 in abundance, %.

Types	Items	Control	S-Control	S-FA100
Phylum	Proteobacteria	47.19 ± 2.34 ^b^	23.09 ± 6.27 ^a^	36.30 ± 7.82 ^ab^
	Bacteroidetes	44.28 ± 3.32 ^b^	16.32 ± 1.04 ^a^	14.42 ± 4.76 ^a^
	Verrucomicrobia	0.08 ± 0.06 ^a^	10.51 ± 1.82 ^b^	6.82 ± 3.20 ^ab^
Class	Chitinophagia	43.91 ± 3.02 ^b^	10.85 ± 4.53 ^a^	13.76 ± 4.83 ^a^
	Alphaproteobacteria	42.06 ± 3.55 ^b^	18.38 ± 5.87 ^a^	25.17 ± 6.96 ^ab^
	Verrucomicrobiae	0.05 ± 0.03 ^a^	10.30 ± 1.90 ^b^	6.48 ± 3.14 ^ab^

Different lowercase letters on the different groups indicate significant differences between groups (Tukey’s test, *p* < 0.05). Data are shown as mean ± SEM (n = 3).

## Data Availability

Data supporting the findings of this study are available in the article and Supplementary Materials. Further inquiries should be addressed to the corresponding author.

## Supplementary Materials

The following supporting information can be downloaded at: https://www.mdpi.com/article/10.3390/ijms27020925/s1.
