# Supplementary material for: Ferulic Acid Attenuates Heat Stress-Induced Hepatic and Intestinal Oxidative Stress and Cholesterol Metabolism Dysregulation in Juvenile Blunt Snout Bream (Megalobrama amblycephala)
Source: Int J Mol Sci. 2026 Jan 16;27(2):925. doi: 10.3390/ijms27020925 (PMC12842258; doi:10.3390/ijms27020925)
Supplement: Supplementary file 1 [file ijms-27-00925-s001.zip › Supplementary Explanation.pdf]

1. Metabolome Analysis Methods

1.1 Metabolites Extraction

Intestinal contents (100 mg) were individually grounded with liquid nitrogen and the homogenate was resuspended with prechilled 80% methanol and 0.1% formic acid by well vortex. Plasma (100 μL) and prechilled methanol (400 μL) were mixed by well vortexing. The samples (the above-mentioned processed intestinal contents and plasma) were incubated on ice for 5 min and then were centrifuged at 15,000 g, 4°C for 5 min. Some of supernatant was diluted to final concentration containing 53% methanol by LC-MS grade water. The samples were subsequently transferred to a fresh Eppendorf tube and then were centrifuged at 15000 g, 4°C for 10 min. Finally, the supernatant was injected into the LC-MS/MS system analysis. Take an equal volume of samples from each experimental sample and mix them together to form the QC sample.

1.2 UHPLC-MS/MS Analysis

UHPLC-MS/MS analyses were performed using a Vanquish UHPLC system (Thermo Fisher, Germany) coupled with an Orbitrap Q ExactiveTM HF mass spectrometer (Thermo Fisher, Germany) in Biozeron Co., Ltd. (Shanghai, China). Samples were injected onto a Hypesil Gold column (100×2.1 mm, 1.9μm) using a 17-min linear gradient at a flow rate of 0.2 mL/min. The eluents for the positive polarity mode were eluent A (0.1% FA in Water) and eluent B (Methanol).The eluents for the negative polarity mode were eluent A (5 mM ammonium acetate, pH 9.0) and eluent B (Methanol). The solvent gradient was set as follows: 2% B, 1.5 min; 2-100% B, 12.0 min; 100% B, 14.0 min; 100-2% B, 14.1 min; 2% B, 17 min. Q ExactiveTM HF mass spectrometer was operated in positive/negative polarity mode with spray voltage of 3.2 kV, capillary temperature of 320°C, sheath gas flow rate of 40 arb and aux gas flow rate of 10 arb (ESI Ion source). The scanning range is set from m/z 100 to 1500. MS/MS secondary scan is data-dependent scans.

1.3 Data processing and metabolite identification

The raw data files generated by UHPLC-MS/MS were processed using the Compound Discoverer 3.1 (CD3.1, Thermo Fisher) to perform peak alignment, peak picking, and quantitation for each metabolite. The main parameters were set as follows: retention time tolerance, 0.2 minutes; actual mass tolerance, 5ppm; signal intensity tolerance, 30%; signal/noise ratio, 3; and minimum intensity, 100000. After that, peak intensities were normalized to the total spectral intensity. The normalized data was used to predict the molecular formula based on additive ions, molecular ion peaks and fragment ions. And then peaks were matched with the mzCloud (https://www.mzcloud.org/), mzVault and MassList database to obtain the accurate qualitative and relative quantitative results. Statistical analyses were performed using the statistical software R (R version R-3.4.3), Python (Python 2.7.6 version) and CentOS (CentOS release 6.6), When data were not normally distributed, normal transformations were attempted using of area normalization method.

1.4 Data Analysis

These metabolites were annotated using the KEGG database (https://www.genome.jp/kegg/pathway.html), HMDBdatabase(https://hmdb.ca/ metabolites) and LIPID Maps databas (http://w lipid). Partial least squares discriminant analysis (PLS-DA) was performed at metaX (aflexible and comprehensive software for ssing tabolomics). The quality of the model was tested using 7-fold cross validation; then, the R2Y (the model's interpretability for the categorical variable Y) and Q2 (the model's predictability) obtained from the cross-validation were used to evaluate the model's effectiveness; R2 and Q2 values above 0.5 are considered good, above 0.4 are acceptable, indicating that the model has good robustness and no overfitting phenomenon. Generally,

when $P \leq 0.05$ and R2 and Q2 are above 0.81, the model is optimal. We applied univariate analysis (t-test) to calculate the statistical significance (*P*-value). The metabolites with VIP > 1 and PDR-adjusted *P*-value< 0.05 and |log2FC| > 1 were considered to be differential metabolites. Volcano plots were used to filter metabolites of interest which based on log2 (Fold Change) and -log10 (p-value) of metabolites. The functions of these metabolites and metabolic pathways were studied using the KEGG database. The metabolic pathways enrichment of differential metabolites was performed, when ratio were satisfied by x/n > y/N, metabolic pathway were considered as enrichment, when PDR-adjusted *P*-value of metabolic pathway < 0.05, metabolic pathway were considered as statistically significant enrichment.

2. Microbial diversity analysis

2.1 DNA extraction and PCR amplification

Microbial DNA was extracted from intestinal contents and plasma samples using the E.Z.N.A.® Soil DNA Kit (Omega Bio-tek, Norcross, GA, U.S.) according to manufacturer's protocols. The V4-V5 region of the bacteria 16S ribosomal RNA gene were amplified by PCR (95 °C for 2 min, followed by 25 cycles at 95 °C for 30 s, 55 °C for 30 s, and 72 °C for 30 s and a final extension at 72 °C for 5 min) using primers 515F 5'-barcode-GTGCCAGCMGCCGCGG-3' and 907R 5'-CCGTCAATTCMTTTRAGTTT-3', where barcode is an eight-base sequence unique to each sample. PCR reactions were performed in triplicate 20 μL mixture containing 4 μL of 5 × FastPfu Buffer, 2 μL of 2.5 mM dNTPs, 0.8 μL of each primer (5 μM), 0.4 μL of FastPfu Polymerase, and 10 ng of template DNA. Amplicons were extracted from 2% agarose gels and purified using the AxyPrep DNA Gel Extraction Kit (Axygen Biosciences, Union City, CA, U.S.) according to the manufacturer's instructions.

2.2 Library Construction and Sequencing

Purified PCR products were quantified by Qubit®3.0 (Life Invitrogen) and every twenty-four amplicons whose barcodes were different were mixed equally. The DNA was fragmented using Ultrasonic instrument. A DNA library was obtained using the NEBNext® Ultra™ DNA Library Prep Kit by unwinding the DNA duplex product from PCR amplification and disrupting the uncircularized DNA molecules. The libraries were finally sequenced using next generation sequencing platform (MGI-G99 or Illumina Miseq) (Shanghai BIOZERON Biotech. Co., Ltd) with PE300 mode according to the standard protocols.

2.3 Processing of sequencing data

Raw fastq files were first demultiplexed using Trimmomatic (http://www.usadellab.org/cms/uploads/supplementary/Trimmomatic) and in-house perlscripts according to the barcode sequences information for each sample with the following criteria: (i) The 300bp reads were truncated at any site receiving an average quality score <20 over a 10 bp sliding window, discarding the truncated reads that were shorter than 50bp. (ii) exact barcode matching, 2 nucleotide mismatch in primer matching, reads containing ambiguous characters were removed. (iii) only sequences that overlap longer than 10 bp were assembled according to their overlap sequence. Reads which could not be assembled were discarded.

OTUs were clustered with 97% similarity cutoff using UPARSE (version 10, http://drive5.com/uparse/) and chimeric sequences were identified and removed using UCHIME. The phylogenetic affiliation of each 16S rRNA gene sequence was analyzed by uclust algorithm (https://github.com/topics/uclust) against the Silva (SSU138.2) 16S rRNA database (http://www.arb-silva.de) using confidence threshold of 80%. After OTUs generation, the analysis of the 16S rDNA data were operated by CFViSA plantform

(http://www.cloud.biomicroclass.com/en/CFViSA), which contained microbiome analysis pipeline and nearly 80 analysis tools spanning simple sequence processing, visualization, and statistics available for the amplicon sequencing data.

2.4 Alpha- and Beta-diversity Analyses

The rarefaction analysisbased on Mothur v.1.21.1 was conducted to reveal the diversity indices, including the Chao1, ACE, Simpson, and Shannon diversity indices. The beta diversity analysis was performed using euclidean distance matrix to principal component analysis (PCA), and bray-curtis or UniFrac distance matrices to compare the results of the principal co-ordinates analysis (PCoA) and non-metric multidimensional scaling (NMDS) with the vegan community ecology package, R-forge (https://r-forge.r-project.org/). Three complementary non-parametric multivariate statistical tests (Adonis, ANOSIM and MRPP) were performed using R vegan package to assess the statistically significant difference of bacterial communities diversity indices between samples. Differences were considered significant at $p < 0.05$. Venn diagrams were drawn using online tool “Draw Venn Diagram” (http://bioinformatics.psb.ugent.be/webtools/Venn) to analyze overlapped and unique OTUs during the treatment processes.
