# Supplementary material for: Ferulic Acid Attenuates Heat Stress-Induced Hepatic and Intestinal Oxidative Stress and Cholesterol Metabolism Dysregulation in Juvenile Blunt Snout Bream (Megalobrama amblycephala)
Source: Int J Mol Sci. 2026 Jan 16;27(2):925. doi: 10.3390/ijms27020925 (PMC12842258; doi:10.3390/ijms27020925)
Supplement: Supplementary file 1 [file ijms-27-00925-s001.zip › Supplementary Tables.pdf]

Supplementary Tables

Table S1 Ingredients and proximate composition of experimental diets (air dry basis, %)

Ingredients	Control	FA100	FA200
Ferulic acid	0.0	0.01	0.02
Soybean meal [a]	23.0	23.0	23.0
Rapeseed meal [a]	15.0	15.0	15.0
Cottonseed meal [a]	10.0	10.0	10.0
Cottonseed protein concentrate [a]	2.0	2.0	2.0
Wheat flour [b]	22.0	22.0	22.0
Rice bran [a]	10.0	10.0	10.0
Soybean oil [b]	4.0	4.0	4.0
Ca $(H_2PO_4)_2$[a]	1.0	1.0	1.0
Vitamin and mineral premix [c]	1.0	1.0	1.0
Vitamin C [c]	1.0	1.0	1.0
Choline chloride (50%) [a]	0.5	0.5	0.5
Mcrocrystalline cellulose [a]	8.5	8.49	8.48
Bentonite [a]	2.0	2.0	2.0
Total	100.0	100.0	100.0
Composition (actual measured values)			
Crude protein	29.15	29.23	29.11
Crude lipid	6.62	6.60	6.65

Note: [a] Provided by Aohua Stock Co., LTD. (Huai'an, China). [b]Purchased from Jingdong Mall, wheat flour is Wudeli brand flour, soybean oil is Fulinmen first class soybean oil. [c]Provided by Wuxi Hanove Animal Health Products Co., Ltd. (Wuxi, China).Vitamin and Mineral Premix (per kg content) : Vitamin A, 1 million IU; Vitamin D3, 200,000 IU; Vitamin E, 10,000 IU; Vitamin K3, 800 mg; Vitamin B1, 2000 mg; Vitamin B2, 2000 mg; Vitamin B6, 2500 mg; Vitamin B12, 10mg; Nicotinamide, 2500 mg; D-pantothenic acid, 3000 mg; Folic acid, 500 mg; D-biotin, 10 mg; Vitamin C, 14000 mg; Inositol, 15000 mg; Copper, 650 mg; Iron, 10500 mg; Manganese, 3500 mg; Zinc, 10750 mg; Magnesium, 17500 mg.

Table S2 Gene primers for RT-PCR

Genes	Genbank Accession no.	Type	Sequence (5'->3')	Annealing temperature	Amplification efficiency	Product length
β-actin	XM_048192430.1	F	TCGTCCACCGCAAATGCTTCTA	62.56	106.6%	152
		R	CCGTCACCTTCACCGTTCCAGT	64.68		
il-6	XM_048203704.1	F	AAGACAACCGCACACTCGAT	59.97	97.2%	122
		R	CTGGGTCTCTTCACGCCTTT	59.96		
il-1β	XM_048181166.1	F	ACCAGCACGACCTTGCAGTG	63.27	108.2%	174
		R	CTGGGATGCATTCGGTTTGA	58.54		
nf-κb	XM_048176853.1	F	AGTCCGATCCATCCGCACTA	60.47	103.2%	85
		R	ACTGGAGCCGGTCATTTCAG	60.04		
caspase-8	XM_048200598.1	F	GCAGATGGACAAGGGAACG	58.53	109.8%	137
		R	TGGCGAAAGGACTGGTAATG	57.89		
bax	XM_048196667.1	F	TTGCTTGAGCTGGTGCAAAC	59.90	105.9%	98
		R	GAGGGGAAGAGCTCAACTGG	59.75		
bcl-2	XM_048179299.1	F	CGTCTACCTGGACAACCACA	59.32	106.5%	190
		R	GCGTTTCTGTGCAATGAGTG	58.32		
xbp1s	XM_048188944.1	F	TCGAGGTGGAAGTCCAAACG	59.97	108.0%	152
		R	TGCATCCGTCAGACACGAAA	59.97		
perk	XM_048204177.1	F	TATTTGTTCAGCGGTGGGCT	59.96	107.3%	206
		R	AAGCCTCCCCCTCTAGGAAG	60.03		
ire1	XM_048190827.1	F	GCTGGAACGCCACATACTCT	60.11	99.7%	103
		R	GTCACCACTAGGCCATCACC	60.11		
bip	XM_048186701.1	F	CCTGGGACAGAAGGTCACAC	59.96	100.4%	133
		R	AGCAGTTGGCTCGTTGATGA	59.96		

hmgcr	XM_048189198.1	F	GCCATCGCTGTCGCTTC	58.90	103.2%	330
		R	CAAGGATGGGAGGCCACAAA	60.25		
abca1	XM_048177071.1	F	AGGACTACTCGGTGTCCCAG	60.32	109.8%	109
		R	ACCGCTGTCTCTTTACGACG	60.11		
apoa1	XM_048188082.1	F	AGCACTACAAGTCAGCAGCC	60.32	101.8%	253
		R	TCCAGCTTGGAACGGATGTC	60.04		
cyp7a1	XM_048186424.1	F	ACCATCCGCTGTTCAGTGAG	60.04	109.0%	203
		R	GAACCCTGCCTCGAACATGA	60.04		
lxrα	XM_048184573.1	F	GCACGTACCTCTACAGTGGC	60.46	102.5%	119
		R	GAGCGTTTGTTGCACTGCTT	60.25		
fxr	XM_048169656.1	F	CAAACATCGCATCCCTCAGGA	60.41	103.7%	230
		R	TGGTGAAAACCTGGGCCGAA	62.29		
aqp8	XM_048171125.1	F	ATGCCATTCAGTCCGCTCAT	59.82	100.1%	139
		R	AGTCCAATGCAGAAGGGAGC	60.03		

F, Forward primer; R, Reverse primer.

Table S3 Valid sequences of intestinal contents of *M. amblycephala*

Groups	Sequences	Average Length(bp)
	13275	1440.71
Control	11957	1438.72
	14101	1442.37
	9708	1435.8
S-Control	11597	1438.53
	14067	1446.8
	20682	1444.43
S-FA100	11162	1434.42
	15291	1444.61

n = 3.

Table S4 Differential metabolites in intestinal contents (S-Control vs Control)

Differential metabolites	log2(FC)	P-value	VIP
Pyrithioxin	9.49	< 0.001	4.33
2'-Deoxyinosine 5'-monophosphate	8.09	0.035	2.37
Terephthalic Acid	7.42	0.027	2.25
N-Benzylformamide	6.23	< 0.001	2.65
3-Methylglutaric acid	6.07	< 0.001	2.52
Cholic acid	6.04	< 0.001	2.83
2,2,5,5-tetramethyl-2,5-dihydro-1H-pyrrole-3-carboxamide	6.00	0.047	1.47
9-chloro-12H-benzo[5,6][1,4]thiazino[2,3-b]quinoxaline	5.74	< 0.001	2.56
2-Keto-4-methylthiobutyric acid	5.71	< 0.001	2.87
Tauroursodeoxycholic acid Dihydrate	5.50	0.001	2.21
Phenylpyruvic acid	5.39	< 0.001	2.56
Dehydroepiandrosterone (DHEA)	5.37	0.010	2.04
7-Ketocholesterol	5.33	< 0.001	3.15
Prostaglandin D2	5.32	< 0.001	2.46
N-Oleoyl Glycine	5.27	< 0.001	2.64
2-oxa-4-azatetracyclo[6.3.1.1~6,10~.0~1,5~]tridecan-3-one	5.27	0.001	2.23
5-(hydroxymethyl)-4-methoxy-2,5-dihydrofuran-2-one	5.20	< 0.001	2.29
Kinetin	5.12	< 0.001	2.39
Xanthosine	4.96	< 0.001	2.58
(6E,10E)-3,7,11,15-tetramethylhexadeca-1,6,10,14-tetraene-3,5,9-triol	4.92	< 0.001	2.46
2-Phenylglycine	4.82	< 0.001	2.18
1-(4-benzylpiperazino)-2-(pyridin-2-ylamino)propan-1-one	4.72	0.001	2.43
20-Hydroxy-(5Z,8Z,11Z,14Z)-eicosatetraenoic acid	4.70	0.043	1.67
2-Ketohexanoic acid	4.67	< 0.001	2.39
5-[(8Z,11Z)-pentadeca-8,11-dien-1-yl]benzene-1,3-diol	4.62	0.004	1.87
5-Methyluridine	4.61	< 0.001	2.05
N-Formylkynurenine	4.60	< 0.001	2.25
3-Methyl-2-oxobutanoic acid	4.59	< 0.001	2.19
Desmethylclozapine	4.50	< 0.001	1.97
N-{4-Methyl-3-[(methylsulfonyl)amino]phenyl}-3-thiophenecarboxamide	4.44	0.001	1.81
L-Cysteine-glutathione gisulfide	4.41	0.023	1.64
ethyl 2-amino-8H-indeno[2,1-b]thiophene-3-carboxylate	4.40	0.002	1.78
5'-Adenylic acid	4.39	< 0.001	2.29
Norbuprenorphine	4.35	0.006	1.53
N-Acetylhistamine	4.28	0.001	1.80
(S)-2-Hydroxybutanoicacid	4.24	< 0.001	2.04
Testosterone sulfate	4.24	0.002	2.21

Differential metabolites	log2(FC)	P-value	VIP
10(11)-EpDPA	4.23	< 0.001	1.98
Orlistat	4.20	< 0.001	1.95
Xanthine	4.19	< 0.001	2.27
11-Prostaglandin F2	4.18	0.040	1.64
Prostaglandin A1 ethyl ester	4.17	< 0.001	2.03
3-Methylthiopropylamine	4.16	0.002	1.89
Quinaldic acid	4.14	< 0.001	2.16
19(20)-DiHDPA	4.12	0.001	1.82
N-Arachidonoyl-L-serine	4.11	0.008	1.45
AL 8810 Methyl ester	4.10	0.001	2.44
Methyl indole-3-acetate	4.05	0.001	1.72
ACar 18:0	3.99	< 0.001	1.74
1-Methylxanthine	3.92	< 0.001	1.97
Thymine	3.89	< 0.001	1.95
Prostaglandin E1 Ethanolamide	3.89	0.001	1.56
Citrulline	3.87	< 0.001	1.93
2-(acetyloxy)-3-amino-1-[1,2-di(acetyloxy)ethyl]-3-oxopropyl acetate	3.86	0.004	1.57
Pantethine	3.85	< 0.001	1.79
4-Methylphenol	3.85	< 0.001	1.74
3-(2-methylpropyl)-octahydropyrrolo[1,2-a]pyrazine-1,4-dione	3.84	0.008	1.27
Syringic acid	3.80	< 0.001	1.72
1,4-dihydroxy-1,4-dimethyl-7-(propan-2-ylidene)-decahydroazulen-6-one	3.74	0.005	1.56
1,2-dihydroxyheptadec-16-yn-4-yl acetate	3.73	0.037	1.24
Lysopg 18:1	3.72	0.024	1.45
3,4-Dihydroxyphenylpropionic acid	3.72	< 0.001	1.71
LysoPC 10:0	3.70	< 0.001	1.80
gamma-Glutamyltyrosine	3.67	< 0.001	1.86
4-Acetamidobutanoic acid	3.66	< 0.001	1.79
Tetrahydrocorticosterone	3.65	< 0.001	1.77
L-Ornithine	3.65	< 0.001	1.82
Benzyl cinnamate	3.65	0.017	1.39
Sodium cholate	3.62	0.001	2.01
Uric acid	3.54	0.001	1.64
ACar 18:1	3.53	0.001	1.59
8-iso-13,14-dihydro-15-keto Prostaglandin F2	3.52	0.004	1.40
D-Proline	3.47	< 0.001	1.69
3-Methylindole	3.47	0.006	1.42
2-Deoxycytidine-5-monophosphoric acid	3.46	0.047	1.15
Lysopc 16:1	3.46	0.002	1.56

Differential metabolites	log2(FC)	P-value	VIP
SDMA	3.44	0.040	1.19
Orotic acid	3.43	0.014	1.39
Urocanic acid	3.43	< 0.001	1.57
Phenethylamine	3.42	0.012	1.47
Stearamide	3.41	0.030	1.64
FAHFA (18:0/20:2)	3.41	0.004	1.50
Lysope 14:0	3.39	< 0.001	1.86
Glycerol 3-phosphate	3.36	0.002	1.55
2-Acetylpyridine	3.35	< 0.001	1.56
Lipoxin B4	3.32	0.008	1.33
Ureidosuccinic acid	3.31	< 0.001	1.70
Calcitriol	3.28	< 0.001	1.60
NSI-189	3.26	< 0.001	1.61
Erdosteine	3.26	0.002	1.30
PPK	3.25	0.001	1.47
2-Aminoadipic acid	3.23	< 0.001	1.55
Phenylacetylglycine	3.23	0.013	1.18
Styrene	3.23	0.009	1.35
L(-)-Carnitine	3.22	< 0.001	1.55
N,N-dimethyl-9H-purin-6-amine	3.21	0.004	1.47
YLH	3.21	0.001	1.35
12-epi Leukotriene B4	3.20	0.003	1.53
Guvacoline	3.19	< 0.001	1.54
UDP-N-acetylglucosamine	3.19	< 0.001	1.62
Norfenefrine	3.19	0.019	1.09
L-Kynurenine	3.16	< 0.001	1.51
5-Phenylvaleric Acid	3.15	< 0.001	1.46
Palmitoleic Acid	3.13	0.004	1.45
Paracetamol	3.11	< 0.001	1.53
18-HEPE	3.11	0.028	1.15
Neopterin	3.08	0.001	1.46
gamma-Glutamylmethionine	3.07	< 0.001	1.51
Desoxycortone	3.07	0.004	1.16
5-chloro-N-(4-morpholinophenyl)-1H-indole-2-carboxamide	3.05	< 0.001	1.56
Acetylcholine	3.03	< 0.001	1.43
Acetyl-L-carnitine	3.03	0.005	1.16
Norverapamil	3.03	0.001	1.29
Cannabidiolic acid	3.03	< 0.001	1.33
ACar 20:1	3.00	0.001	1.28
4-(methylthio)-6-phenyl-2-(3-pyridyl)pyrimidine-5-carbonitrile	2.99	< 0.001	1.49
Palmitoylcarnitine	2.97	0.017	1.09

Differential metabolites	log2(FC)	P-value	VIP
D-Ribulose 5-phosphate	2.97	0.014	1.29
D-Glucosamine	2.96	< 0.001	1.41
7-hydroxy-3-phenyl-4H-chromen-4-one	2.95	< 0.001	1.45
Nonadecanoic acid	2.95	0.003	1.28
YMK	2.94	0.026	1.07
THC	2.91	< 0.001	1.46
Oxoadipic Acid	2.91	0.025	1.02
alpha-Ketoglutaric acid	2.89	0.001	1.70
beta-Nicotinamide mononucleotide	2.88	0.016	1.07
4-Pregnen-17alpha,20alpha-Diol-3-One	2.84	< 0.001	1.45
8(9)-DiHET	2.82	< 0.001	1.46
5-Hydroxytryptophan	2.81	< 0.001	1.44
Inosine 5'-Monophosphate	2.80	0.002	1.21
11,12-Epoxy-(5Z,8Z,11Z)-icosatrienoic acid	2.79	0.005	1.42
3b,7b-Dihydroxy-5-androsten-17-one	2.79	0.003	1.12
2-Linoleoyl glycerol	2.79	< 0.001	1.49
1-Palmitoyl-Sn-Glycero-3-Phosphocholine	2.78	0.003	1.27
15-OxoEDE	2.78	< 0.001	1.23
2-(Dimethylamino)Guanosine	2.77	< 0.001	1.35
Maslinic acid	2.77	0.001	1.15
3-Acetoxyurs-12-en-23-oic acid	2.75	0.006	1.09
2-(Formylamino)Benzoic Acid	2.73	< 0.001	1.33
acetyl phosphate	2.73	0.013	1.17
Pseudouridine	2.73	0.002	1.16
Ergocristine	2.72	0.028	1.11
17(S)-HpDHA	2.72	< 0.001	1.45
L-(+)-Citrulline	2.70	< 0.001	1.39
3-hydroxyquinuclidine-3-carbonitrile hydrochloride	2.70	0.002	1.32
N-(8-methyl-8-azabicyclo[3.2.1]oct-3-yl)-4-nitrobenzamide	2.69	0.024	1.00
Pantothenic acid	2.69	0.001	1.22
Prostaglandin F2alpha	2.67	< 0.001	1.24
(1E,4E)-1,5-bis(4-methoxyphenyl)penta-1,4-dien-3-one	2.67	< 0.001	1.23
N-Acetyl-D-alloisoleucine	2.65	< 0.001	1.24
15(S)-HpEPE	2.64	0.008	1.19
3-(3-pyridinyl)propanoic acid	2.63	0.001	1.16
3-Chloro-L-tyrosine	2.63	< 0.001	1.26
2-Oxobutyric acid	2.62	0.001	1.26
O-Acetyl-L-carnitine	2.61	0.002	1.10
Vatalanib dihydrochloride	2.60	0.007	1.04
2-Hydroxymyristic acid	2.60	0.002	1.21
Pleuromutilin	2.57	0.008	1.11

Differential metabolites	log2(FC)	P-value	VIP
All-Trans-13,14-Dihydroretinol	2.55	0.009	1.18
Robenidine	2.55	0.009	1.03
4-Hydroxy-3-methylbenzoic acid	2.55	< 0.001	1.15
Pentadecanoic acid	2.53	0.012	1.06
Thromboxane B2	2.53	0.003	1.06
1-[4-(1-adamantyl)phenoxy]-3-piperidinopropan-2-ol hydrochloride	2.49	0.005	1.49
Malic acid	2.48	0.001	1.14
N-acetyl-D-glucosamine	2.47	0.006	1.20
N6-Acetyl-L-lysine	2.45	0.005	1.05
Arachidonoyl amide	2.43	0.035	1.10
Phe-Pro	2.43	< 0.001	1.15
Tropolone	2.41	0.002	1.07
AICA ribonucleotide	2.40	< 0.001	1.13
9-(3-O-Methylpentofuranosyl)-1,9-dihydro-6H-purin-6-one	2.39	0.001	1.19
N-Acetylsphingosine	2.37	0.020	1.23
Indole-3-acrylic acid	2.37	0.001	1.05
15-Deoxy-12,14-prostaglandin D2	2.36	< 0.001	1.16
D-(+)-Camphor	2.35	0.008	1.12
Octadeca-11E,13E,15Z-trienoic acid	2.34	0.003	1.01
Dihydroroseoside	2.34	0.001	1.28
Stearoyl ethanolamide	2.34	0.001	1.40
Aldosterone	2.32	< 0.001	1.14
RNK	2.32	0.003	1.07
9-HpOTrE	2.29	0.012	1.05
4-(hydroxymethyl)benzoic acid	2.28	0.001	1.01
13(S)-HOTrE	2.26	0.002	1.32
L-5-Hydroxytryptophan	2.26	0.001	1.14
2'-O-Methyluridine	2.26	< 0.001	1.13
N,N-Dimethylaniline	2.26	< 0.001	1.05
Xanthurenic acid	2.24	0.001	1.36
4-Hydroxybutyric acid (GHB)	2.24	0.001	1.18
Hypoxanthine	2.23	0.002	1.20
23-Norcholic acid	2.23	0.002	1.03
2'-Deoxyuridine	2.20	0.001	1.18
(+/-)9-HpODE	2.20	< 0.001	1.11
ACar 12:1	2.16	0.004	1.08
Glycodeoxycholic acid	2.16	0.003	1.24
Docosahexaenoic acid	2.15	0.002	1.08
DL-methionine sulfoxide	2.12	< 0.001	1.03
Sulfoacetic acid	2.11	< 0.001	1.05

Differential metabolites	log2(FC)	P-value	VIP
Dl-2-Amino-3-phosphonopropionic acid	2.07	0.001	1.03
(R)-3-Hydroxy myristic acid	2.05	< 0.001	1.03
13,14-dihydro Prostaglandin F1	2.05	< 0.001	1.01
(+/-)8(9)-DiHET	2.03	0.004	1.12
Biopterin	2.03	0.001	1.17
Guanosine 5'-diphosphate (GDP)	2.03	< 0.001	1.01
2-Deoxyuridine	1.99	0.004	1.04
Butylparaben	1.98	< 0.001	1.04
1,4-Cyclohexanedicarboxylic acid	1.98	0.033	1.04
L-Aspartic acid	1.95	0.001	1.00
FIBF-d7	1.94	0.010	1.03
all-cis-4,7,10,13,16-Docosapentaenoic acid	1.94	0.005	1.13
TQH	1.90	0.006	1.14
N-{6-[4-(tert-butyl)phenoxy]-3-pyridinyl}-N'-(2-thienyl)urea	1.86	0.007	1.01
PC (18:2/18:2)	1.70	0.040	1.11
N6-Succinyl Adenosine	1.69	0.039	1.03
MAG (18:4)	1.65	0.013	1.10
FAHFA (22:5/18:2)	1.47	0.019	1.04
2-Arachidonyl Glycerol ether	1.40	0.013	1.07
Adrenic acid	1.39	0.019	1.15
Docosatrienoic acid	1.32	0.031	1.01
FAHFA (22:5/20:2)	1.31	0.025	1.27
5'-Deoxy-5'-(Methylthio)Adenosine	-1.34	0.040	1.02
(9cis)-Retinal	-1.34	0.025	1.24
N1-[4-hydroxy-6-(methoxymethyl)pyrimidin-2-yl]acetamide	-1.43	0.010	1.52
Cetirizine N-oxide	-1.49	0.017	1.02
3-[(4-hydroxyphenyl)methyl]-octahydropyrrolo[1,2-a]pyrazine-1,4-dione	-1.53	0.039	1.00
PLK	-1.56	0.014	1.11
Sedanolide	-1.78	0.014	1.34
Gly-Val	-1.78	0.003	1.06
N-{4-[(2R,3R)-3-(Hydroxymethyl)-5-oxo-2-morpholinyl]phenyl}acetamide	-1.80	0.012	1.15
(+/-)11(12)-EET	-1.82	0.039	1.19
3-(propan-2-yl)-octahydropyrrolo[1,2-a]pyrazine-1,4-dione	-1.82	0.002	1.00
4-Hydroxy-L-Glutamic Acid	-1.83	0.004	1.05
4-[4-phenyl-3,6-dihydro-1(2H)-pyridinyl]butanoic acid hydrochloride	-1.86	0.022	1.31
2-Furoic acid	-1.88	0.002	1.06
13,14-dihydro-15-keto-tetranor Prostaglandin E2	-1.91	0.007	1.66
2'-O-Methyl-5-methylcytidine	-1.91	0.007	1.48

Differential metabolites	log2(FC)	P-value	VIP
LPA 22:6	-1.92	< 0.001	1.00
Valylproline	-1.95	0.006	1.16
N1-[4-(acetylamino)phenyl]-2,2-dimethylcyclopropane-1-carboxamide	-1.98	0.001	1.01
Sebacic acid	-1.99	0.014	1.33
EPK	-1.99	0.003	1.02
PLH	-1.99	< 0.001	1.07
5-Methoxysalicylic acid	-2.00	< 0.001	1.02
Epitestosterone	-2.00	0.010	1.59
N1-(2-oxoazepan-3-yl)-N2-(3-pyridylmethyl)ethanediamide	-2.04	< 0.001	1.03
7-hydroxy-4-[(2-pyridylthio)methyl]-2H-chromen-2-one	-2.04	0.003	1.28
2-(3,4-dimethoxyphenyl)-N-(4-morpholinophenyl)acetamide	-2.05	0.004	1.00
4-methoxy-6-(prop-2-en-1-yl)-2H-1,3-benzodioxole	-2.05	< 0.001	1.02
D-(-)-Fructose	-2.11	< 0.001	1.11
Wampetin	-2.16	0.003	1.03
Ala-Ile	-2.16	0.001	1.17
Celestolide	-2.17	0.001	1.19
Methyl EudesMate	-2.18	< 0.001	1.12
(5-L-Glutamyl)-L-Amino Acid	-2.19	0.003	1.16
Gamma-Glu-Leu	-2.19	0.002	1.24
D-Threose	-2.19	0.001	1.08
D-(-)-Lyxose	-2.19	< 0.001	1.07
Agmatine	-2.23	0.021	1.07
4-Thiouridine	-2.23	0.001	1.06
VQH	-2.24	< 0.001	1.13
Delta-Tridecalactone	-2.25	0.041	1.18
tetranor-PGFM	-2.27	< 0.001	1.29
D-(-)-Mannitol	-2.27	0.001	1.03
gamma-Glutamylleucine	-2.27	0.001	1.27
Folinic acid	-2.27	< 0.001	1.16
5-Phenyl-N-(4-(trifluoromethyl)phenyl)oxazol-2-amine	-2.27	< 0.001	1.26
D-Mannitol 1-phosphate	-2.29	0.001	1.22
L-Alanyl-L-proline	-2.29	0.001	1.37
Gluconic acid	-2.30	0.005	1.22
L-Threonic acid	-2.30	0.002	1.29
tert-Butyl N-[1-(aminocarbonyl)-3-methylbutyl]carbamate	-2.30	< 0.001	1.20
Maltotriose	-2.38	0.003	1.15
1,4-dihydroxyheptadec-16-en-2-yl acetate	-2.40	< 0.001	1.15
N2-(2,4-dimethylphenyl)-1,3-benzothiazol-2-amine	-2.40	0.008	1.08
Val-Ser	-2.41	0.003	1.37

Differential metabolites	log2(FC)	P-value	VIP
1-acetyl-N-(6-chloro-1,3-benzothiazol-2-yl)-4-piperidinecarboxamide	-2.41	0.002	1.19
2-oxopiperidine-3-carbohydrazide	-2.42	< 0.001	1.14
Sulfamethoxazole hydroxylamine	-2.43	< 0.001	1.34
Phenylpropiolic acid	-2.43	< 0.001	1.18
D-(-)-Quinic acid	-2.44	< 0.001	1.23
(2-anilino-4-methyl-1,3-thiazol-5-yl)(4-methoxyphenyl)methanone	-2.44	0.002	1.11
N-{[2-(2-thienyl)-1,3-thiazol-4-yl]methyl}benzamide	-2.44	< 0.001	1.26
2-Isobutyl-3-methoxypyrazine	-2.46	< 0.001	1.21
6-Hydroxymelatonin	-2.46	0.009	1.48
6-Hydroxycortisol	-2.46	0.002	1.07
S-Adenosyl-L-methionine	-2.47	0.011	1.15
16H-dinaphtho[2,1-d:1,2-g][1,3]dioxocine	-2.50	< 0.001	1.30
Ala-Val	-2.50	0.002	1.38
1,5-Anhydro-D-glucitol	-2.54	0.001	1.29
6-Sialyllactose	-2.56	< 0.001	1.31
Betaine	-2.59	0.002	1.22
4-(3,4-dihydro-2H-1,5-benzodioxepin-7-yl)-2-methyl-1,3-thiazole	-2.62	0.004	1.07
Riboflavin-5-phosphate	-2.63	< 0.001	1.44
N1-methyl-5-methoxy-2-({2-[(methylamino)carbonyl]phenyl}thio)benzamide	-2.64	< 0.001	1.43
Liquiritigenin	-2.64	< 0.001	1.35
Daidzein	-2.65	< 0.001	1.46
hydroxyphenylpyruvate	-2.66	< 0.001	1.36
benzaldehyde 1-[4-(3-pyridyl)pyrimidin-2-yl]hydrazone	-2.67	< 0.001	1.39
Methyl beta-D-galactopyranoside	-2.68	< 0.001	1.33
Feruloyl Putrescine	-2.69	< 0.001	1.36
Homotaurine	-2.71	< 0.001	1.42
N~5~-(pyridin-2-ylmethyl)-1H-1,2,4-triazole-3,5-diamine	-2.72	< 0.001	1.40
D-Ala-D-Ala	-2.72	< 0.001	1.35
Genistein 4'-O-glucuronide	-2.74	< 0.001	1.33
Methyl-beta-galactopyranoside	-2.76	< 0.001	1.43
Boc-beta-cyano-L-alanine	-2.79	< 0.001	1.44
N-Acetyl-DL-tryptophan	-2.79	0.001	1.65
Gly-Phe	-2.80	< 0.001	1.40
Sildenafil N-oxide	-2.81	0.014	1.08
2-(2-amino-3-methylbutanamido)-3-phenylpropanoic acid	-2.83	0.003	1.16
1-(6-hydroxy-4,7-dimethoxybenzo[b]furan-5-yl)ethan-1-one	-2.83	< 0.001	1.45
Trehalose	-2.85	< 0.001	1.44

Differential metabolites	log2(FC)	P-value	VIP
3-hydroxy-1,5-diphenylpentan-1-one	-2.89	< 0.001	1.54
3-(3,4-Dihydroxyphenyl)-2-Methylalanine	-2.89	0.008	1.12
Corey Lactone Diol	-2.89	0.006	1.26
5-Hydroxyindole-2-carboxylic acid	-2.89	< 0.001	1.70
diethyl 2-{[2-(2,4-dichlorophenoxy)anilino]methylidene}malonate	-2.97	0.001	2.09
6-Methylflavone	-2.98	0.001	1.41
Porphobilinogen	-2.98	< 0.001	1.62
(2E)-6-hydroxy-2-methyl-6-(4-methylphenyl)hept-2-enoic acid	-2.99	< 0.001	1.56
Folic acid	-3.01	< 0.001	1.80
Dihydroorotic acid	-3.02	0.003	1.32
L-(+)-Arabinose	-3.03	< 0.001	1.43
Obscurolide A1	-3.04	< 0.001	1.57
Mupirocin	-3.07	0.005	1.28
Glycitein	-3.10	< 0.001	1.68
N,5-Bis(3-(trifluoromethyl)phenyl)oxazol-2-amine	-3.11	< 0.001	1.57
3-(2,4-dichlorophenyl)pentanedioic acid	-3.13	< 0.001	1.59
(-)-Caryophyllene oxide	-3.14	< 0.001	1.72
Genistein	-3.14	< 0.001	1.69
Malonic acid	-3.14	0.001	1.74
MQH	-3.14	< 0.001	1.46
5-Hydroxyindole-3-acetic acid	-3.16	< 0.001	1.62
Biotin	-3.16	0.017	1.26
Alanyltyrosine	-3.16	0.003	1.34
N-(4-morpholinophenyl)-4-(1H-pyrazol-1-yl)benzamide	-3.17	< 0.001	1.81
3-(3,4,5-trimethoxyphenyl)propanoic acid	-3.19	< 0.001	1.57
Resorcinol	-3.21	< 0.001	1.55
N,N'-di[4-(2,6-dimethylmorpholino)phenyl]thiourea	-3.21	< 0.001	1.86
Kinetin 9-riboside	-3.23	< 0.001	1.50
Isoferulic acid	-3.25	< 0.001	1.68
5,8-dihydroxy-10-methyl-5,8,9,10-tetrahydro-2H-oxecin-2-one	-3.26	< 0.001	1.72
2-[6-(1H-benzo[d]imidazol-2-yl)-2-pyridyl]-1H-benzo[d]imidazole	-3.28	< 0.001	1.71
6-methyl-7-nitro-2,3-dihydro-1,4-benzodioxine	-3.29	< 0.001	1.79
Genistin	-3.32	< 0.001	1.71
7-chloro-2-methyl-3-(4-pyridylmethyl)-3,4-dihydroquinazolin-4-one	-3.32	< 0.001	1.67
Aspartylphenylalanine	-3.36	< 0.001	1.78
2-(1H-benzimidazol-2-yl)-N-[4-(benzyloxy)phenyl]benzamide	-3.36	< 0.001	1.63
HET0016	-3.44	0.001	1.66

Differential metabolites	log2(FC)	P-value	VIP
N-[3-(1H-imidazol-1-yl)propyl]-5-methoxy-1H-indole-2-carboxamide	-3.52	< 0.001	1.72
Cryptotanshinone	-3.55	< 0.001	1.97
D-Glucarate	-3.55	< 0.001	1.70
Monolaurin	-3.57	0.003	1.51
4-methyl-5-phenoxy-6-piperidino-2-(trifluoromethyl)pyrimidine	-3.57	< 0.001	1.78
Bilirubin	-3.58	< 0.001	1.76
4-(benzoylamino)-3-hydroxybutanoic acid	-3.68	< 0.001	1.94
HRH	-3.68	< 0.001	1.82
Toradol	-3.70	< 0.001	1.80
6-Oxycodol N-oxide	-3.72	< 0.001	1.84
Pyridoxine	-3.73	< 0.001	1.74
Naringenin	-3.73	< 0.001	2.00
Trehalose	-3.74	< 0.001	1.94
N-(2-hydroxy-2-phenylethyl)-N'-(2-thienyl)urea	-3.78	< 0.001	1.91
3',5,7-Trihydroxy-4'-methoxyflavanone	-3.79	< 0.001	1.74
5-(benzylsulfonyl)-4-chloro-2-methyl-2,3-dihydropyridazin-3-one	-3.79	< 0.001	1.89
Isorhamnetin	-3.84	< 0.001	2.00
1-(3-acetyl-2,4,6-trihydroxyphenyl)ethan-1-one	-3.87	< 0.001	1.96
Apocynin	-3.88	< 0.001	1.96
Phenylglyoxylic acid	-3.90	< 0.001	1.95
Esculin	-3.92	< 0.001	1.98
Ethyl chrysanthemumate	-3.95	< 0.001	1.98
1H-indol-3-yl(pyridin-2-yl)methanol	-3.96	< 0.001	1.90
Gentisic acid	-3.99	< 0.001	2.02
Glycerol 1-hexadecanoate	-4.00	0.046	1.38
D-Saccharic acid	-4.00	< 0.001	1.99
ethyl 1-(3-nitro-2-thienyl)piperidine-4-carboxylate	-4.01	0.005	1.48
3-[3-(methylthio)anilino]-1,3-dihydroisobenzofuran-1-one	-4.02	< 0.001	2.02
15-epi Cloprostenol	-4.05	< 0.001	2.15
Lactose	-4.09	0.001	2.17
3-Hydroxyanthranilic Acid	-4.12	< 0.001	1.97
2-[(5-anilino-4-phenyl-4H-1,2,4-triazol-3-yl)thio]acetic acid	-4.13	< 0.001	2.09
Maltopentaose	-4.19	< 0.001	2.24
4,5-Dicaffeoylquinic acid	-4.19	< 0.001	2.03
Leukotriene C4	-4.25	< 0.001	2.07
3-(4-hydroxy-3-methoxyphenyl)propanoic acid	-4.26	< 0.001	2.13
12-Oxo leukotriene B4	-4.27	< 0.001	1.95
2,4-dihydroxyheptadec-16-en-1-yl acetate	-4.27	0.001	1.99
Poncirin	-4.35	< 0.001	2.01

Differential metabolites	log2(FC)	P-value	VIP
Spiculisporic Acid	-4.41	< 0.001	2.29
Dl-3,4-Dihydroxymandelic Acid	-4.42	< 0.001	2.14
Sucrose	-4.45	< 0.001	2.17
Trehalose 6-phosphate	-4.49	< 0.001	2.28
MAG (18:3)	-4.53	0.030	1.53
8-{[(3S)-3-(1H-Benzimidazol-2-yl)-1-pyrrolidinyl]sulfonyl}quinoline	-4.60	< 0.001	2.34
6-methyl-5-nitroquinoline	-4.61	< 0.001	2.29
3-Methoxybenzaldehyde	-4.62	< 0.001	2.47
Maltotetraose	-4.65	< 0.001	3.02
1-[(3,5-dimethylisoxazol-4-yl)sulfonyl]piperidine	-4.65	0.002	1.94
D-(-)-Ribose	-4.68	< 0.001	2.34
N-(p-Coumaroyl) serotonin	-4.74	< 0.001	2.38
Deoxyinosine	-4.80	< 0.001	2.80
Ferulic acid	-4.83	< 0.001	2.56
Glucobrassicin	-4.83	< 0.001	2.45
Oleanolic acid	-4.92	< 0.001	2.53
Adipamide	-4.97	< 0.001	2.50
3-(3-Methoxyphenyl)propionic acid	-4.98	< 0.001	2.59
13,14-Dihydro prostaglandin E1	-5.00	0.048	1.65
Catechin	-5.14	< 0.001	2.58
trans-Aconitic acid	-5.21	< 0.001	2.67
4-Hydroxyglucobrassicin	-5.23	< 0.001	2.46
Quercetin-3-D-glucoside	-5.30	< 0.001	2.61
Trifolin	-5.35	< 0.001	2.77
2,3-dihydroxypropyl 12-methyltridecanoate	-5.36	0.009	2.17
3-hydroxy-2-[5-nitro-2-(1-pyrrolidinyl)benzyl]propanenitrile	-5.36	< 0.001	2.68
5,7-dihydroxy-3-(4-hydroxyphenyl)-4H-chromen-4-one	-5.39	0.030	1.77
Kaempferol	-5.43	< 0.001	2.74
NNK	-5.44	< 0.001	2.75
D-Raffinose	-5.45	< 0.001	3.31
5-(9H-xanthen-9-yl)-1,3,4-oxadiazole-2-thiol	-5.45	< 0.001	2.82
Aflatoxin G1	-5.45	< 0.001	2.97
Glycitin	-5.48	< 0.001	2.75
1,5,8-Trihydroxy-9-oxo-9H-xanthen-3-yl beta-D-glucopyranoside	-5.49	< 0.001	2.86
Chlorogenic acid	-5.49	< 0.001	3.09
Vitamin B2	-5.52	< 0.001	2.77
Testosterone undecanoate	-5.52	< 0.001	2.71
Vitexin	-5.62	< 0.001	2.85
Stercobilin	-5.66	< 0.001	2.59

Differential metabolites	log2(FC)	P-value	VIP
methyl 4-methyl-2-oxo-2H-pyran-6-carboxylate	-5.74	< 0.001	2.93
3-Methoxy prostaglandin F1	-5.82	< 0.001	2.66
Stachyose	-5.84	< 0.001	3.87
Verbascose	-5.84	< 0.001	3.26
DL-3,4-Dihydroxyphenyl glycol	-6.07	< 0.001	3.04
Gluconapin	-6.10	< 0.001	3.84
Dehydroascorbic acid	-6.17	< 0.001	3.13
Quinic acid	-6.35	< 0.001	2.97
Rutin	-6.44	< 0.001	3.26
N1-(6-methyl-4-oxo-3,4-dihydroquinazolin-2-yl)-4-nitrobenzamide	-6.45	< 0.001	3.52
Quercetin	-6.67	< 0.001	3.39
Daidzin	-6.71	< 0.001	3.32
4-((5-(4-Nitrophenyl)oxazol-2-yl)amino)benzonitrile	-6.98	< 0.001	4.37
ethyl 2-thioxo-4-(trifluoromethyl)-1,2-dihydropyrimidine-5-carboxylate	-7.66	< 0.001	3.89
Sinapinic acid	-8.99	< 0.001	4.57

Notes: Differential metabolite screening threshold: VIP ≥ 1 & FDR-adjusted *P*-value ≤ 0.05 & |log2FC| ≥ 1; VIP: Variable importance in the projection values for the PLS-DA model, P-value: T-test for metabolite abundance in both S-Control and Control groups, FC: mean abundance ratios of the two groups S-Control and Control. n = 6.

Table S5 Differential metabolites in intestinal contents (S-FA100 vs S-Control)

Differential metabolites	log2(FC)	P-value	VIP
Thromboxane B1	3.63	0.023	2.74
4-methyl-6-phenyl-5,6-dihydro-2H-pyran-2-one	3.51	0.046	2.20
Gemifloxacin	3.38	0.021	2.25
2'-Deoxyadenosine	3.31	0.036	2.18
N-Acetylneuraminic acid	3.28	0.016	2.53
1-(3-ethyl-2,4-dihydroxy-6-methoxyphenyl)butan-1-one	3.23	0.002	3.62
1-acetyl-N-(6-chloro-1,3-benzothiazol-2-yl)-4-piperidinecarboxamide	3.08	0.015	2.31
N~5~-(pyridin-2-ylmethyl)-1H-1,2,4-triazole-3,5-diamine	3.03	0.033	1.76
Dihydrokawain	3.02	0.013	2.08
1-(2,4-dihydroxyphenyl)-2-(3,5-dimethoxyphenyl)propan-1-one	2.93	0.024	1.86
¦Á-Linolenoyl ethanolamide	2.90	0.041	1.69
FLK	2.68	0.043	2.96
HRH	2.65	0.011	1.90
N-Acetyl-5-aminosalicylic acid	2.65	0.008	1.84
5'-Deoxy-5'-(Methylthio)Adenosine	2.58	0.043	2.37
5-Methylcytosine	2.47	0.037	1.80
2-(1-{2-[(3-furylmethyl)amino]-2-oxoethyl}cyclohexyl)acetic acid	2.46	0.046	1.69
8-(2,3-dihydroxy-3-methylbutyl)-7-methoxy-2H-chromen-2-one	2.46	0.035	1.60
L-cysteine	2.46	0.023	1.90
Estrone sulfate	2.36	0.033	1.61
geranyl pp	2.23	0.026	1.54
benzaldehyde 1-[4-(3-pyridyl)pyrimidin-2-yl]hydrazone	2.20	0.005	1.95
3-methyl-5-oxo-5-(4-toluidino)pentanoic acid	2.17	< 0.001	2.08
3'-Hydroxystanozolol	2.16	0.039	2.06
4-methoxy-9-(3-methylbut-2-en-1-yl)-7H-furo[3,2-g]chromen-7-one	2.12	0.022	1.43
3-(3,4,5-trimethoxyphenyl)propanoic acid	2.11	0.022	1.38
4-[2-(2-oxo-1-imidazolidinyl)ethyl]-1lambda~6~,4-thiazinane-1,1-dione	1.98	0.016	1.78
Acetaminophen glucuronide	1.97	0.049	1.58
2-benzyl-6-hydroxy-2-azabicyclo[2.2.2]octan-3-one	1.97	0.017	2.39
8-iso-13,14-dihydro-15-keto Prostaglandin F2¦Á	1.93	0.048	1.96
4-(2,3-dihydro-1,4-benzodioxin-6-yl)butanoic acid	1.91	0.019	2.64
5-acetyl-2,6-dimethyl-1,2,3,4-tetrahydropyridin-4-one	1.86	0.032	1.33
N1-(2,3-dihydro-1,4-benzodioxin-2-ylmethyl)-2,2-dimethylpropanamide	1.83	0.001	1.69
Oxymatrine	1.80	0.003	1.62
Monobutyl phthalate	1.77	0.016	1.91
4-Butylresorcinol	1.75	0.004	1.42
gamma-Nonanolactone	1.74	0.045	1.22

Differential metabolites	log2(FC)	P-value	VIP
1-(4-bromophenyl)-2-phenylethan-1-one	1.70	< 0.001	1.80
N1-(3-cyano-4,6-diphenyl-2-pyridyl)-4-methylbenzamide	1.68	0.042	1.73
N-Acetylaspartic acid	1.68	0.007	1.57
Levalbuterol	1.68	0.004	1.60
5,8-dihydroxy-10-methyl-5,8,9,10-tetrahydro-2H-oxecin-2-one	1.66	0.044	1.39
1-(6-hydroxy-4,7-dimethoxybenzo[b]furan-5-yl)ethan-1-one	1.66	0.034	1.35
4-[(2-cyclohex-1-enylethyl)amino]-2H-chromen-2-one	1.66	0.023	1.88
N-¦Á-L-Acetyl-arginine	1.65	0.021	1.23
7,8-Dihydrofolate	1.64	0.013	1.77
Corchorifatty acid F	1.64	0.003	1.36
5,5-dimethyl-3-morpholinocyclohex-2-en-1-one	1.62	0.010	1.49
3-(2-naphthyl)-5-(trifluoromethyl)-1H-pyrazole	1.61	0.044	1.25
Piceatannol	1.60	0.045	1.14
19(R)-hydroxy Prostaglandin E2	1.52	0.036	1.13
Ecgonine	1.51	0.025	1.48
Gibberellin A4	1.49	0.019	1.70
(2R)-2-[(2R,5S)-5-[(2S)-2-hydroxybutyl]oxolan-2-yl]propanoic acid	1.48	0.016	1.09
13,14-dihydro-15-keto-PGD2	1.47	0.022	1.04
N-Acetyl-aspartic acid	1.46	0.008	1.28
N~5~-(1,3,5-trimethyl-1H-pyrazol-4-yl)-1H-1,2,4-triazole-3,5-diamine	1.46	0.008	1.36
5-Hydroxytryptophan	1.45	0.036	1.04
5-(benzylsulfonyl)-4-chloro-2-methyl-2,3-dihydropyridazin-3-one	1.45	0.023	1.11
2,3-dinor Prostaglandin E1	1.45	0.004	1.56
Oxoadipic Acid	1.42	0.040	1.91
cis-2-Decenoic acid	1.38	0.010	1.09
Beta-Muricholic acid	1.36	0.045	1.02
L(-)-Carnitine	1.36	0.027	1.16
ELK	1.35	0.038	2.39
AKB48 N-(4-fluorobenzyl) analog	1.35	0.009	1.23
2-(1-adamantyl)-1-morpholinoethan-1-one	1.35	0.003	1.26
Mestranol	1.32	0.046	1.40
6,15-diketo-13,14-dihydro Prostaglandin F1¦Á	1.32	0.005	1.17
Royal jelly acid	1.32	0.002	1.14
Thymine	1.31	0.023	1.33
Prostaglandin F3¦Á	1.29	0.047	1.39
ACar 7:0	1.28	0.018	1.49
3,4-Dihydroxyphenylpropionic acid	1.26	0.035	1.38
ALK	1.26	0.016	3.80
2,5-bis(4-hydroxy-3-methoxyphenyl)-3,4-dimethyloxolan-3-ol	1.25	0.041	1.02

Differential metabolites	log2(FC)	P-value	VIP
Adenosine 5'-Diphosphate	1.23	0.021	1.18
D-¦Á-Hydroxyglutaric acid	1.19	0.037	1.14
Virginiamycin	1.17	0.049	1.17
4-Amino-5-imidazolecarboxamide	1.17	0.048	1.00
Sildenafil-d3	1.17	0.027	1.38
4,5-diphenyl-2-(3,4,5-trimethoxyphenyl)-1H-imidazole	1.14	0.014	1.01
UR-144 N-(5-hydroxypentyl) ¦Â-D-glucuronide	1.12	0.030	1.27
Prostaglandin D3	1.12	0.026	1.03
3-[(4-chlorophenyl)thio]-1-phenylprop-2-en-1-one	1.10	0.036	1.07
3',5'-Cyclic dAMP	1.09	0.008	1.17
1H-indol-3-yl(pyridin-2-yl)methanol	1.08	0.024	1.03
Sulfoacetic acid	1.07	0.003	1.06
N-{[(2R,4S,5R)-5-Ethyl-1-azabicyclo[2.2.2]oct-2-yl]methyl}-2-furamide	1.02	0.049	1.02
gamma-Glutamylcysteine	1.00	0.021	1.20
4-Oxoproline	1.00	0.015	1.15
Benzoic acid	-1.00	0.049	5.87
Lysopc 14:0	-1.02	0.044	1.70
THC	-1.02	0.035	1.36
ACar 18:2	-1.04	0.043	1.93
2,5-Dihydroxybenzaldehyde	-1.06	0.048	4.11
Linolelaidic Acid (C18:2N6T)	-1.06	0.017	1.04
4-Pregnen-17alpha,20alpha-Diol-3-One	-1.07	0.033	1.54
Heptanoic acid	-1.08	0.047	4.37
L-Fucose	-1.08	0.046	4.18
L-(+)-Tartaric acid	-1.09	0.046	4.81
methylamino-l-alanine	-1.13	0.045	3.36
Nonanoic acid	-1.13	0.045	4.53
Capric acid	-1.13	0.044	3.86
Prostaglandin K1	-1.13	0.032	1.43
Albendazole sulfone	-1.14	0.043	1.06
Glycodeoxycholic acid	-1.14	0.038	1.53
Coenzyme Q2	-1.14	0.028	1.38
2-Arachidonyl Glycerol ether	-1.28	0.007	1.34
8-Bromoguanosine	-1.28	0.001	1.22
16-Hydroxyhexadecanoic acid	-1.29	0.032	1.58
2-Anisic acid	-1.32	0.038	4.02
FAHFA (18:2/3:0)	-1.42	0.047	1.90
Docosatrienoic acid	-1.44	0.028	2.15
MAG (18:4)	-1.46	0.032	2.51
Adrenic acid	-1.53	0.032	2.23

Differential metabolites	log2(FC)	P-value	VIP
Octadeca-11E,13E,15Z-trienoic acid	-1.54	0.038	1.13
Methyltestosterone	-1.54	0.036	1.63
Lysopc 16:2 (2N Isomer)	-1.54	0.011	1.62
(+/-)9,10-dihydroxy-12Z-octadecenoic acid	-1.58	0.026	1.62
MAG (18:2)	-1.58	< 0.001	1.52
Milbemectin A3	-1.63	0.049	1.29
trans-10-Heptadecenoic Acid	-1.64	0.037	1.84
Theophylline	-1.65	0.030	4.52
FAHFA (18:1/3:0)	-1.65	0.016	2.28
2-Linoleoyl glycerol	-1.73	0.003	1.62
Ergocalciferol	-1.77	0.011	1.49
11(Z),14(Z)-Eicosadienoic Acid	-1.87	0.021	2.31
Docosapentaenoic acid	-1.87	0.018	2.36
11(E)-Eicosenoic Acid	-1.89	0.049	1.92
Cholecalciferol	-1.97	0.023	1.72
1-Palmitoyl-Sn-Glycero-3-Phosphocholine	-2.17	0.014	1.96
N1-(4H-1,2,4-triazol-4-yl)-4-nitrobenzamide	-2.20	0.007	1.71
FAHFA (22:4/18:0)	-2.42	0.012	2.67
FAHFA (20:2/22:4)	-2.47	0.005	2.47
FAHFA (20:2/20:3)	-2.61	0.016	1.81
Lysopg 18:1	-3.33	0.040	2.62

Notes: Differential metabolite screening threshold: VIP ≥ 1 & FDR-adjusted *P*-value ≤ 0.05 & |log2FC| ≥ 1; VIP: Variable importance in the projection values for the PLS-DA model, P-value: T-test for metabolite abundance in both S-FA100 and S-Control groups, FC: mean abundance ratios of the two groups S-FA100 and S-Control. n = 6.

Table S6 Differential metabolites in plasma (S-Control vs Control)

Differential metabolites	log2(FC)	P-value	VIP
PLK	4.10	< 0.001	3.40
2-Aminoadipic acid	3.90	0.002	2.82
Cystathionine	3.85	< 0.001	3.13
(R)-3-Hydroxy myristic acid	3.76	< 0.001	3.33
Creatine	3.64	< 0.001	3.07
Imidazolelactic acid	3.50	0.001	3.52
Acetylcholine	3.47	< 0.001	3.15
L(-)-Carnitine	3.44	< 0.001	3.11
Valylproline	3.37	< 0.001	2.90
Desthiobiotin	3.22	< 0.001	2.73
Prostaglandin D2	3.04	< 0.001	2.82
11-Dehydro thromboxane B2	2.85	< 0.001	2.54
Propionylcarnitine	2.53	0.001	2.28
Prolylleucine	2.52	< 0.001	2.12
L-Cystathionine	2.41	0.003	1.76
Acetyl-L-carnitine	2.39	< 0.001	2.13
Pilocarpine	2.32	0.004	1.82
4-Hydroxybutyric acid (GHB)	2.19	< 0.001	2.03
1-butyl-2-methyl-4-nitro-1H-imidazole	2.16	0.001	2.02
N6,N6,N6-Trimethyl-L-lysine	2.11	< 0.001	1.87
Creatine phosphate	2.04	0.002	1.74
4-oxo-5-phenylpentanoic acid	1.98	0.006	1.46
¦Â-Estradiol-17¦Â-glucuronide	1.94	0.006	1.60
Xanthosine	1.92	0.004	1.66
Taurine	1.81	< 0.001	1.55
Hexanoylcarnitine	1.73	< 0.001	1.59
1,2-Dihydro-1,2-naphthalenediol	1.69	< 0.001	1.57
Phosphocreatine	1.67	0.005	1.49
1-Methylhistidine	1.67	0.001	1.57
1,4-Dihydro-1-Methyl-4-Oxo-3-Pyridinecarboxamide	1.64	0.001	1.53
Cytosine	1.62	< 0.001	1.46
Uric acid	1.60	0.010	1.63
ACar 20:4	1.56	< 0.001	1.43
2-Hydroxy-2-methylbutanoic acid	1.48	< 0.001	1.36
Xanthine	1.46	0.039	1.15
Oxoadipic Acid	1.45	0.020	1.06
O-Acetylserine	1.44	< 0.001	1.30
N-Acetylaspartic acid	1.36	0.045	1.31
Adenosine 3'5'-cyclic monophosphate	1.34	0.002	1.17
Oxaceprol	1.33	0.002	1.11
trans-Aconitic acid	1.29	0.036	1.44
2,5-Furandicarboxylic acid	1.29	< 0.001	1.18
TQH	1.28	< 0.001	1.17
10-Hydroxydecanoic acid	1.27	0.007	1.10
ACar 14:1	1.27	0.001	1.20
L-Glutamic acid	1.26	0.002	1.10

Differential metabolites	log2(FC)	P-value	VIP
ACar 16:1	1.26	< 0.001	1.13
Palmitoylcarnitine	1.23	< 0.001	1.14
ACar 10:1	1.20	< 0.001	1.10
2-Furoic acid	1.18	0.049	1.10
4-Butylresorcinol	1.18	< 0.001	1.11
2-Anisic acid	1.11	0.008	1.12
4-Phenylbutyric acid	1.11	0.001	1.07
N-Acetyl-L-leucine	1.06	0.001	1.02
Riboflavin	-1.07	< 0.001	1.03
11-Epiprostaglandin E1	-1.08	< 0.001	1.00
Guanosine monophosphate	-1.09	< 0.001	1.00
LPC 20:5	-1.10	< 0.001	1.03
PC (20:3/22:6)	-1.14	0.004	1.12
Methyltestosterone	-1.14	< 0.001	1.06
FRH	-1.14	< 0.001	1.04
11(Z),14(Z),17(Z)-Eicosatrienoic acid	-1.15	0.040	1.00
PC (18:3/22:6)	-1.15	0.012	1.18
LPC 22:4	-1.16	< 0.001	1.02
Taurochenodeoxycholic Acid (sodium salt)	-1.19	0.012	1.16
3-(3,4-Dihydroxyphenyl)-2-Methylalanine	-1.19	< 0.001	1.03
LPC 18:3	-1.19	< 0.001	1.10
PE (16:1e/20:4)	-1.23	0.007	1.01
5-(isopentylthio)-1,3,4-thiadiazol-2-amine	-1.23	0.002	1.10
L-Tyrosine	-1.23	0.001	1.06
PC (20:4e/2:0)	-1.24	0.001	1.06
Salicylic acid	-1.26	< 0.001	1.18
4-Hydroxybenzaldehyde	-1.28	0.014	1.26
LPC 20:3	-1.28	0.001	1.11
Asparagine	-1.30	0.001	1.14
PC (18:5e/6:0)	-1.33	< 0.001	1.19
(¡À)11(12)-EET	-1.37	< 0.001	1.19
N-Acetylornithine	-1.38	0.025	1.48
(¡À)12(13)-DiHOME	-1.39	< 0.001	1.30
PC (14:1e/2:0)	-1.41	0.003	1.14
5-Hydroxyindole	-1.43	< 0.001	1.30
Acipimox	-1.45	0.001	1.33
Lysops 22:6	-1.45	< 0.001	1.28
Lysope 16:0	-1.47	0.015	1.06
(3beta,9xi)-3-(beta-D-Glucopyranosyloxy)-14-hydroxycard-20(22)-enolide	-1.49	0.016	1.47
GlcADG (16:0-20:3)	-1.49	0.002	1.35
2-[(1,3-thiazol-2-ylamino)methylidene]malononitrile	-1.51	0.001	1.38
PC (22:6e/2:0)	-1.52	< 0.001	1.34
L-Ascorbic acid 2-sulfate	-1.54	0.006	1.45
LPE 20:5	-1.54	< 0.001	1.40
N-lactoyl-phenylalanine	-1.60	0.001	1.36
Mag (18:1)	-1.64	0.001	1.51
PC (18:5e/2:0)	-1.64	< 0.001	1.39

Differential metabolites	log2(FC)	P-value	VIP
Hexadecanedioic acid	-1.66	< 0.001	1.50
N-Acetyl-L-tyrosine	-1.67	< 0.001	1.51
8Z,11Z,14Z-Eicosatrienoic acid	-1.69	0.001	1.46
2,3-dinor Prostaglandin E1	-1.70	< 0.001	1.56
5-Phenyl-N-(4-(trifluoromethyl)phenyl)oxazol-2-amine	-1.70	< 0.001	1.60
2-Arachidonoyl glycerol	-1.70	< 0.001	1.48
FAHFA (18:1/20:3)	-1.80	< 0.001	1.67
PC (18:4e/3:0)	-1.81	0.020	1.44
LPE 20:4	-1.82	< 0.001	1.57
MAG (18:3)	-1.83	0.032	1.33
Vitamin A	-1.84	0.043	1.23
Adrenic acid	-1.84	0.010	1.47
MAG (18:2)	-1.84	0.002	1.59
N-Acetylhistidine	-1.84	< 0.001	1.56
L-Ascorbate	-1.87	0.005	1.50
N,5-Bis(3-nitrophenyl)oxazol-2-amine	-1.89	< 0.001	1.75
Arachidonic acid	-1.90	0.004	1.54
S-Adenosyl-methionine	-1.91	< 0.001	1.77
LPI 18:1	-1.94	0.014	1.48
13(S)-HOTrE	-1.95	< 0.001	1.78
Prostaglandin H2	-1.97	< 0.001	1.83
Palmitoleic Acid	-1.98	< 0.001	1.80
1,2-dihydroxyheptadec-16-yn-4-yl acetate	-1.98	< 0.001	1.76
(+/-)9-HpODE	-2.03	< 0.001	1.86
Docosapentaenoic acid	-2.12	0.001	1.72
Eicosapentaenoic acid	-2.12	< 0.001	1.77
2-hydroxy-6-[(8Z,11Z)-pentadeca-8,11,14-trien-1-yl]benzoic acid	-2.18	0.012	1.70
8-iso Prostaglandin A2	-2.18	< 0.001	2.01
Sucrose	-2.19	< 0.001	1.98
DLK	-2.20	0.001	1.78
Equol	-2.21	< 0.001	2.00
1,3,7-trimethyl-2,3,6,7-tetrahydro-1H-purine-2,6-dione	-2.22	< 0.001	2.06
LPC 18:4	-2.39	< 0.001	2.12
11,12-Epoxy-(5Z,8Z,11Z)-icosatrienoic acid	-2.44	0.010	1.84
D-Galactosamine	-2.44	< 0.001	2.23
Methyl beta-D-galactopyranoside	-2.45	< 0.001	2.31
N1-[4-hydroxy-6-(methoxymethyl)pyrimidin-2-yl]acetamide	-2.45	< 0.001	2.19
Methyl EudesMate	-2.55	< 0.001	2.10
Milbemectin A3	-2.57	< 0.001	2.46
Prostaglandin K2	-2.60	< 0.001	2.31
L-Methionine sulfoxide	-2.73	< 0.001	2.50
Prostaglandin D3	-2.76	< 0.001	2.58
9-HpOTrE	-2.76	< 0.001	2.45
FAHFA (22:4/20:3)	-2.79	0.003	2.22
11-Deoxy prostaglandin F2¦Â	-2.80	< 0.001	2.57
FAHFA (18:2/20:4)	-3.06	< 0.001	2.54
N1-isopropyl-2-(1H-2-pyrrolylcarbonyl)-1-hydrazinecarboxamide	-3.14	< 0.001	2.66
5¦Á-Pregnan-3,20-dione	-3.24	0.042	2.10

Differential metabolites	log2(FC)	P-value	VIP
PC (18:3/18:3)	-3.50	0.005	2.42
Pregnenolone	-3.74	0.006	2.65
N-[2-(6-amino-9H-purin-9-yl)ethyl]-N-(2-furylmethyl)amine	-3.89	< 0.001	3.55

Notes: Differential metabolite screening threshold: VIP ≥ 1 & FDR-adjusted *P*-value ≤ 0.05& |log2FC| ≥ 1; VIP: Variable importance in the projection values for the PLS-DA model, P-value: T-test for metabolite abundance in both S-Control vs Control groups, FC: mean abundance ratios of the two groups S-Control and Control. n = 6.

Table S7 Differential metabolites in plasma (S-FA100 vs S-Control)

Differential metabolites	log2(FC)	P-value	VIP
1,2-dihydroxyheptadec-16-yn-4-yl acetate	2.44	0.027	3.38
11-Deoxy prostaglandin F2¦Â	2.06	0.033	3.06
Prostaglandin K2	2.02	0.016	3.04
9-HpOTrE	1.97	0.027	2.86
Prostaglandin D3	1.91	0.047	2.89
2,3-dinor Prostaglandin E1	1.56	0.011	2.67
PC (20:1/18:2)	1.51	0.013	2.63
Hexadecanedioic acid	1.47	0.011	2.51
2-hydroxy-6-[(8Z,11Z)-pentadeca-8,11,14-trien-1-yl]benzoic acid	1.43	0.021	2.64
ACar 16:1	1.30	0.023	2.12
11(Z),14(Z),17(Z)-Eicosatrienoic acid	1.12	0.018	2.29
PC (18:5e/16:4)	1.09	0.001	2.11
PC (14:0e/19:1)	1.04	< 0.001	2.02
Pyrogallol	-1.15	0.044	1.77
PC (18:5e/19:1)	-1.22	0.038	7.15

Notes: Notes: Differential metabolite screening threshold: VIP ≥ 1 & FDR-adjusted *P*-value ≤ 0.05 & |log2FC| ≥ 1; VIP: Variable importance in the projection values for the PLS-DA model, P-value: T-test for metabolite abundance in both S-FA100 and S-Control groups, FC: mean abundance ratios of the two groups S-FA100 and S-Control groups. n = 6.
